# Toward Long-Acting Injectable Antidepressants: Drug-Delivery Strategies, Clinical Considerations, and Regulatory Barriers

**DOI:** 10.3390/pharmaceutics18101323

**Published:** 2026-10-19

**Authors:** Haripriya Koppisetti, Weranga Rajapaksha, Harsh P. Nijhawan, Yunmei Song, Souha Youssef, Sanjay Garg

**Affiliations:** Centre for Pharmaceutical Innovation, College of Health, Adelaide University, Adelaide, SA 5000, Australia; haripriya.koppisetti@adelaide.edu.au (H.K.); weranga.rajapakshagedara@adelaide.edu.au (W.R.); harsh.nijhawan@adelaide.edu.au (H.P.N.); may.song@adelaide.edu.au (Y.S.); souha.youssef@adelaide.edu.au (S.Y.)

**Keywords:** long-acting injectables (LAIs), antidepressants, sustained-release formulations, patient adherence, polymer-based drug delivery systems, lipid-based drug delivery systems

## Abstract

Long-acting injectable (LAI) systems may offer an alternative approach to antidepressant treatment by reducing reliance on daily oral medication, supporting adherence, limiting pharmacokinetic fluctuations, and maintaining therapeutic drug exposure over extended periods. These potential advantages may be particularly relevant for patients with recurrent depression or difficulty sustaining regular oral treatment. However, converting conventional antidepressants into clinically useful LAIs remains challenging because many drugs require substantial maintenance doses, have formulation-limiting physicochemical properties, and may require dose adjustment or prompt discontinuation in response to adverse effects. This review evaluates the feasibility of established and emerging depot technologies, including polymeric microspheres, in situ-forming depots, lipid- and oil-based systems, hydrogels, and hybrid or stimulus-responsive platforms, for sustained antidepressant delivery. It compares these approaches according to drug-loading capacity, release mechanisms, achievable dosing interval, injection-volume constraints, manufacturing scalability, sterilization, product robustness, safety, local tissue tolerability, and regulatory maturity. Major translational barriers include the high total drug mass required for many antidepressants, acceptable injection-volume limits, solvent and excipient safety, injection-site reactions, limited reversibility after administration, pharmacokinetic drug-interaction risk, difficulty developing predictive in vitro–in vivo correlations and demanding regulatory expectations for complex injectable products. The review also considers opportunities for coordinated long-acting combination therapy, personalized patient selection, digital adherence monitoring, and emerging biomaterials. Overall, successful LAI antidepressant development will require careful matching of the drug molecules, delivery platform, pharmacokinetic target, and intended patient population. Early products are most likely to rely on established depot technologies and prior confirmation of oral efficacy and tolerability before long-term administration.

## 1. Introduction

According to the World Health Organization (WHO), one in every eight people in the world are living with a mental disorder. A mental disorder is a clinically significant disturbance in an individual’s cognition, behavior, or emotional regulation, mainly due to impairment or distress [1]. Mental disorder is a broad term that covers psychological disabilities, impairment in the functioning of certain regions of the brain, or stress-associated disorders. Mental disorders include anxiety, depression, bipolar disorder, post-traumatic stress disorder, schizophrenia, eating disorders, disruptive behavior and dissocial disorders, and neurodevelopmental disorders [2]. As per the survey in 2019, around 970 million people are living with mental disorders, with anxiety and depression being the most common mental disorders [3].

**Figure 1:**
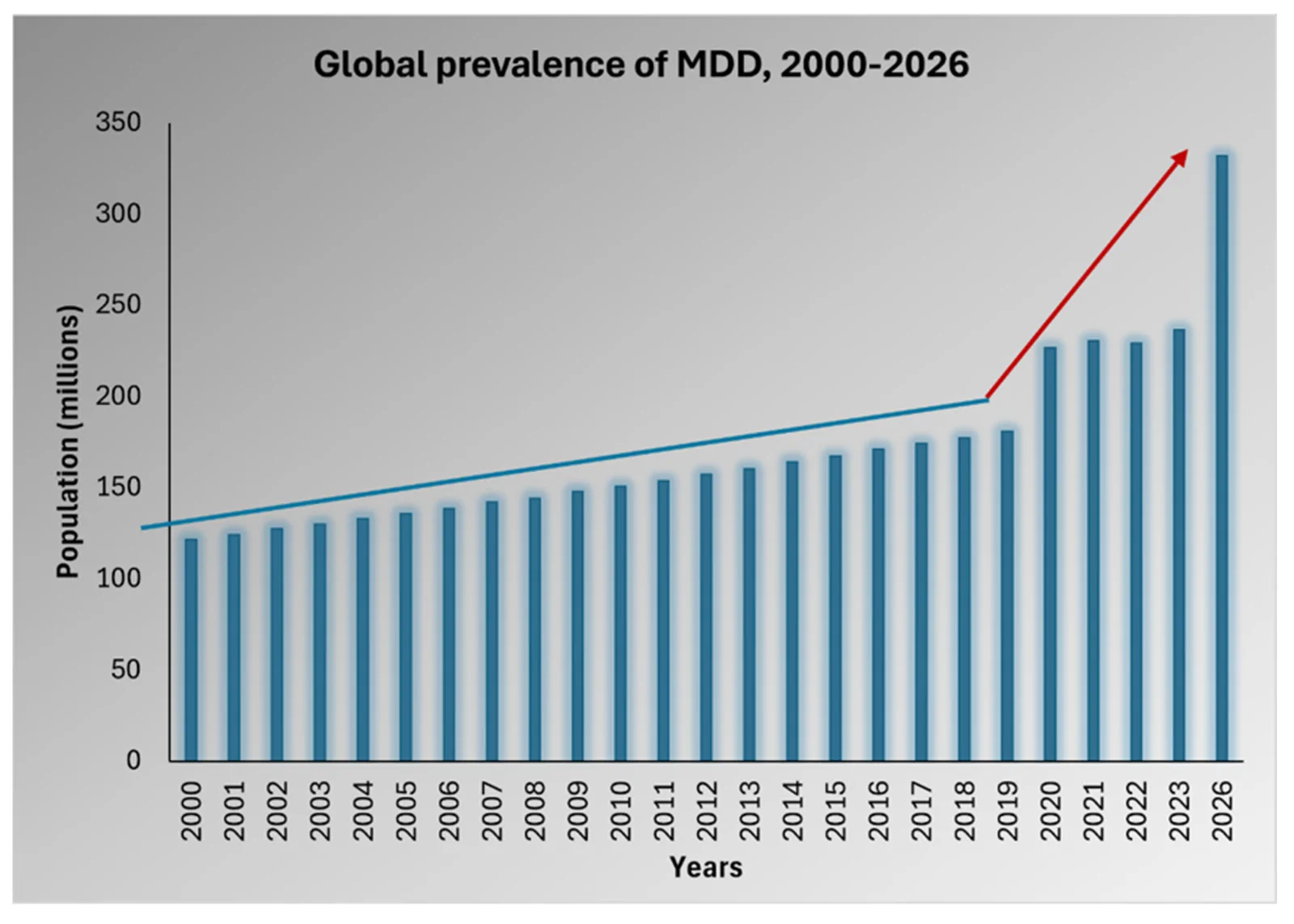
Global prevalence of MDD from 2000 to 2026 as per IHME and WHO. The graph was created by the authors using data on results from the Global Burden of Disease Collaborative Network’s Global Burden of Disease Study 2023 (GBD 2023), based in the United States (using the Institute for Health Metrics and Evaluation (IHME) 2024 Tool), using prevalence and number metrics, for both sexes, all ages, and global location (https://vizhub.healthdata.org/gbd-results/. Accessed on 09 August 2026) [4].

According to the World Health Organization, depression is defined as **“***the most common mental disorder observed in both adults and children, which involves loss of pleasure, depressive mood, or loss of interest in activities for long periods.*” [1]. As per the latest statistics by the Institute of Health Metrics and Evaluation (IHME), approximately 332 million people are suffering from depression globally, and more than 700,000 people die by suicide due to depression every year; it is considered the third leading cause of death in 15–29-year-olds [1]. As per the data from the National Institute of Mental Health (NIMH), 21 million adults in the United States are suffering from depression, with the majority of risk in young adults aged 18 to 25 years [2]. One in six people experience depression at some time in their life. According to the recent reports of the National Study of Mental Health and Wellbeing, in Australia, 42.9% of people aged 15–85 years experienced mental illness at some time in their life, and 38.8% among them had a 12-month mental disorder [3]. Following the COVID-19 pandemic, the number increased to 28% for anxiety and depression (detailed statistics of prevalence are mentioned in Figure 1).

Depression can vary from mild to severe, with symptoms including feelings of sadness, guilt, loss of interest, hopelessness, low self-esteem, low concentration, sleep disturbances, and increased or decreased appetite [5]. These symptoms may worsen over time, affecting an individual’s mental and physical health and social behavior, leading to suicidal thoughts. Several factors like abuse (could be physical or emotional), lack of social support, living alone, low self-esteem, lack of confidence, criticism, life events (like trauma, death of loved ones, long-term unemployment), drug or alcohol abuse, medical conditions (like obesity or any chronic illness) and usage of certain medications, as well as heredity in some cases, contribute to the progression of the disease [6]. Based on the severity and symptoms, depression is classified further into specific conditions like major depressive disorder, bipolar disorder, cyclothymic disorder, dysthymic disorder, seasonal affective disorder, and perinatal depression, as mentioned in Table 1.

Although psychological factors seem to be responsible for depression, there is an underlying neurological alteration that plays a key role in the progression of the disease. As per in vivo and postmortem studies by Nemeroff et al., serotonin (5-HT) transporters and receptors have a major role in the progression of depression and anxiety [13]. 5-HT is the neurotransmitter crucial for the development of emotional circuits in the brain, and alterations in its levels cause imbalance, leading to mood disturbances [14]. Brain imaging and studies carried out in patients suffering from depression revealed neurofunctional alterations in emotion-related areas of the brain, particularly in the 5-HT region, and decreased neurotransmitter signaling cascade due to reduced cyclic AMP (cAMP) response element-binding protein (CREB) [15]. CREB is a nuclear protein produced in the post-synaptic neurons as a result of protein kinase A (PKA), and protein kinase C (PKC) is responsible for regulating neurological signaling [16].

**Figure 2:**
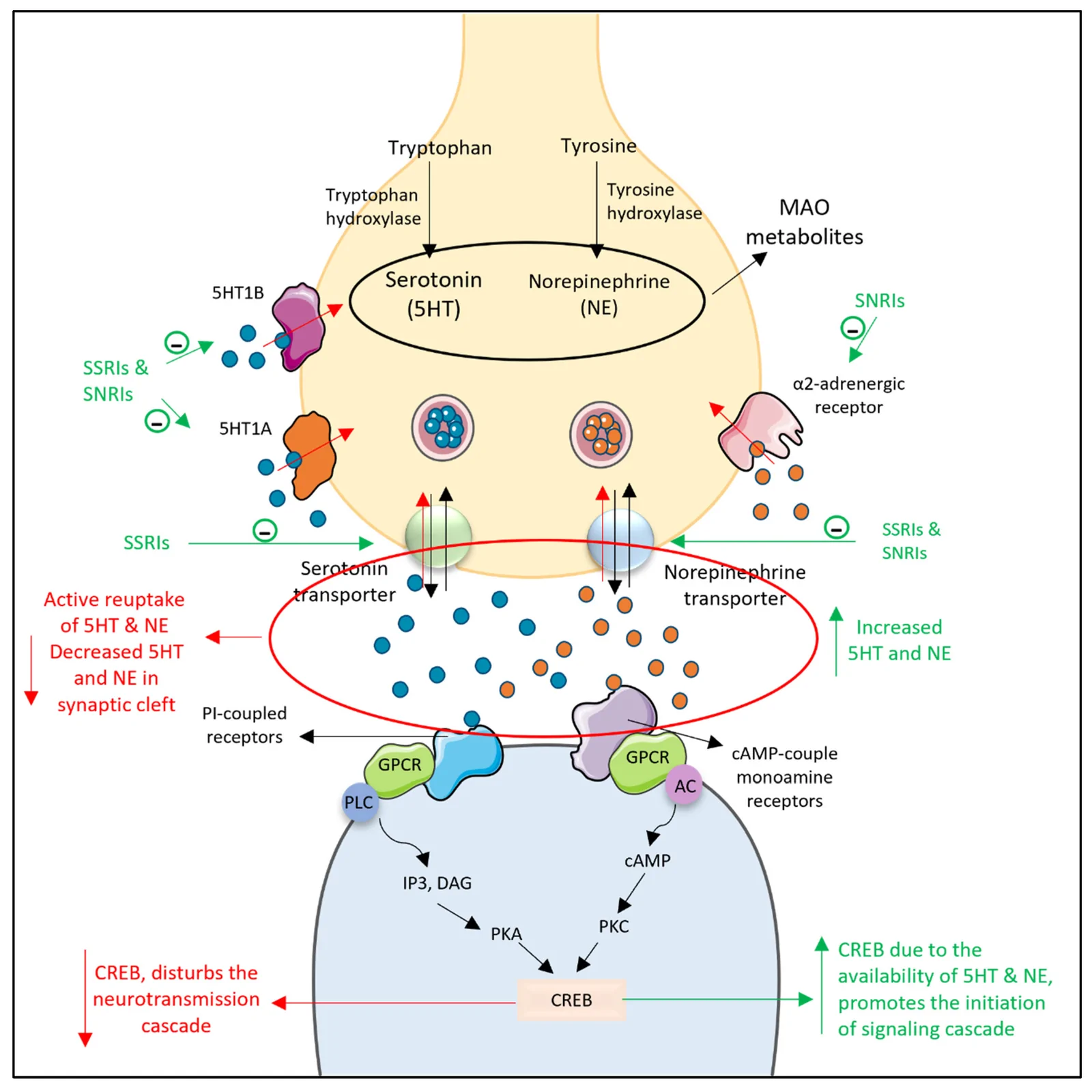
Diagrammatic representation of mechanism involved in depression; 5-HT: 5-hydroxy tryptamine (serotonin); NE: norepinephrine; 5-HT1A and B: 5-hydroxy tryptamine receptors A and B; GPCR: G-protein coupled receptors; PLC: Phospholipase C; AC: Adenyl cyclase; IP3: Inosine phosphate 3; PI-coupled receptors: Phospho-Inosine (PI)-coupled monoamine receptors; cAMP: Cyclic Adenyl monophosphate; CREB: Cyclic AMP response element binding protein; SSRIs: selective serotonin reuptake inhibitors; SNRIs: serotonin and norepinephrine reuptake inhibitors. The red arrows represent active reuptake and low levels of 5-HT and NE, and the green lines represent the action of antidepressants to increase 5-HT and NE (illustration created using Servier Medical Art, https://smart.servier.com/, licensed under CC BY 4.0; https://creativecommons.org/licenses/by/4.0/).

The pathway (as shown in Figure 2) contributing to CREB begins with the synthesis of neurotransmitters serotonin (5-HT) and norepinephrine (NE) in presynaptic neurons from tryptophan and tyrosine, respectively, via tryptophan hydroxylase and tyrosine hydroxylase. The synthesized 5-HT and NE are also referred to as monoamine oxidase metabolites due to their chemical nature. 5-HT and NE are released into the synaptic cleft via 5-HT and NE transporter channels, which then bind to the receptors present on pre- and post-synaptic neurons. 5-HT and NE have an affinity towards 5-HT receptors, adrenergic receptors present pre-synaptically, and G-protein coupled receptors (GPCR) present post-synaptically. On post-synaptic neurons, these neurotransmitters bind to Phospho-Inosine (PI)-coupled monoamine receptors and cAMP-coupled monoamine receptors, resulting in the activation of phospholipase C (PLC) and adenyl cyclase (AC), respectively. PLC activates Inosine phosphate 3 (IP3) and Diacylglycerol (DAG), finally resulting in the generation of PKC. Similarly, AC activates cAMP, resulting in the generation of PKA, which, along with PKC, activates CREB [17].

When the brain is exposed to long-term stress, 5-HT and NE transporters and receptors present on presynaptic neurons malfunction, increasing the reuptake of 5-HT and NE. This results in low availability of 5-HT and NE in the synaptic cleft, altering the binding with GPCR on post-synaptic neurons. This results in reduced PKA and PKC activation, which leads to decreased CREB for the initiation of the signaling cascade [18]. Antidepressants (each class of antidepressant is discussed in the following sections) are designed such that they increase the concentration of 5-HT and NE in the synaptic cleft by inhibiting their reuptake, making them available for GPCR binding and increasing CREB levels [19].

### 1.1. Current Treatments and Their Challenges

**Figure 3:**
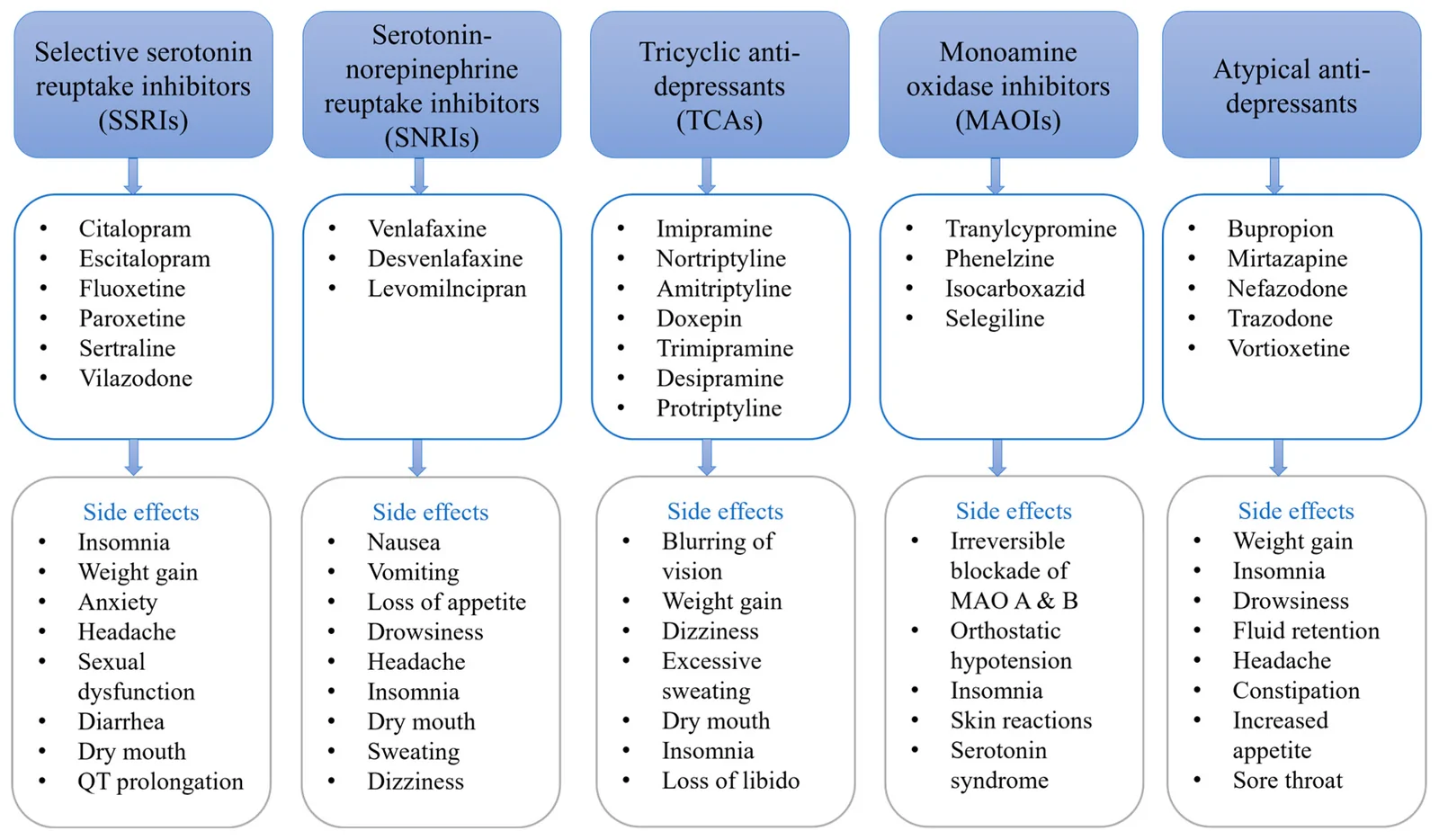
List of different classes of antidepressants along with their side effects.

According to the WHO and the National Institute of Mental Health, depression can be managed by psychotherapy, using antidepressant drugs, or both, depending on the severity. Treatment involving drugs is preferred for patients who are in severe stages or early moderate stages of depression or who have other mental disorders associated with depression, like anxiety or schizophrenia [1,2]. Although there is a pathophysiology explaining the mechanism of depression, studies are still ongoing to understand it in depth. From the various studies taking pre-clinical and clinical evidence, it is clear that 5-HT and NE play a key role in the development of depression and are identified as target sites for treatment [20]. According to the WHO and the American Psychological Association (APA), antidepressants are classified as selective serotonin reuptake inhibitors (SSRIs), selective serotonin and norepinephrine reuptake inhibitors (SNRIs), tricyclic antidepressants (TCAs), monoamine oxidase inhibitors (MAOIs), and atypical antidepressants, as mentioned in Figure 3 [21].

Although many types of oral antidepressants are available, findings from the Sequenced Treatment Alternatives to Relieve Depression (STAR*D) study show that around 10–15% of individuals with depression do not return to the treatment after their first evaluation, and between 20 and 50% of patients discontinue the therapy within six months. And among those who continue the treatment, 50% exhibit poor adherence, often due to personal, societal, or economic burdens [22]. These burdens are intensified by the chronic and recurring nature of the disorder, which further affects functioning and overall quality of life [23]. While oral antidepressants have proven effective in both acute and ongoing treatment, their success heavily relies on consistent long-term use. Unfortunately, early discontinuation and poor adherence remain widespread, and many patients stop taking their medications within a few months due to the side effects, lack of perceived benefit, or uncertainty about pharmacotherapy [24]. Such discontinuation increases the likelihood of relapses, recurrence, and withdrawal symptoms. These patterns undermine the preventive benefits of antidepressants and highlight the need for treatment models that reduce vulnerability to inconsistent use [25].

### 1.2. Long-Acting Injectable (LAI) Platform

Oral dosage forms are the most preferred and widely used route of administration due to their ease of administration and low cost. Although it is the most patient-compliant route, it is not the optimal dosage form for drugs with poor bioavailability, poor absorption, low permeability, and extensive first-pass metabolism [26]. Alternatively, the parenteral route is a more suited model for such drugs, which can enhance the bioavailability, surpass the first-pass metabolism, and show a faster onset of action [27].

LAI formulations are emerging as a favorable strategy to address the persistent gaps in the pharmacological management of MDD. Although LAIs are well-established in schizophrenia and bipolar disorder, their application in depression remains limited. And depression is a chronic, relapsing illness that often requires long-term, continual pharmacotherapy, posing challenges in patient adherence. This non-adherence is driven by multiple factors, including side effects, delayed onset of action, stigma, forgetfulness and lack of interest due to depressive episodes. These limitations highlight the need for delivery systems capable of maintaining stable, sustained delivery systems.

LAI formulations offer several advantages that directly address these challenges. By providing sustained therapeutic plasma levels over extended periods (days, weeks or months), LAIs minimize the peak–trough fluctuations, reduce the burden of daily dosing and improve adherence. They also reduce the likelihood of discontinuation, which is particularly relevant for antidepressants associated with withdrawal symptoms. From a clinical perspective, LAIs may support better continuity of care, better relapse rates, and improved functional outcomes. LAI formulations can be developed as colloidal systems, liposomes, lipid nanoparticles, polymeric nanoparticles, microspheres, microemulsions, implants, or in situ-forming depot systems. These modified systems can provide the drug for a longer duration in the body, minimizing dosage frequency. This is especially suitable for patients with neurological disorders like depression, Alzheimer’s, Parkinson’s, and epilepsy [28]. LAIs have been extensively studied in the field of antipsychotic treatments (as mentioned in Table 2).

Taken together, these considerations underscore the unmet need for systematically developed LAI antidepressant formulations and provide the rationale for the present review to critically evaluate available depot technologies and their translational potential in mood disorders [37]. However, the current review discusses the evidence relevant to LAI antidepressant development under three distinct levels. Firstly, we consider direct evidence, including studies evaluating antidepressants in sustained-release or LAI formulations, including formulation, pharmacokinetic, and preclinical safety studies. Indirect platform evidence includes marketed or investigational LAI products containing antipsychotics, peptides, analgesics, endocrine therapies or other drugs, which inform manufacturing feasibility, release mechanism, safety and regulatory considerations but do not establish antidepressant efficacy. Lastly, conceptual extrapolation refers to hypotheses that apply established LAI principles to antidepressant candidates based on their physicochemical and pharmacokinetic properties.

The success rate of LAI antipsychotics such as risperidone, paliperidone and olanzapine in schizophrenia and bipolar disorder provides a standard example of improved adherence, fewer relapses and hospitalizations, and, in some contexts, reduced mortality compared with oral therapy [38]. Their adoption has been driven by advances in depot technologies, clearer recognition of non-adherence in psychotic disorders, and evolving attitudes among clinicians and patients [39]. In contrast, despite the similar adherence challenges in depression and anxiety disorders and robust evidence that premature discontinuation increases the risk of relapses, LAI development for antidepressants is still lacking. This gap in research could be due to limitations including higher dose requirements for antidepressants, uncertainties about acceptable injection volumes and excipients, and the historical perception of depression as less “severe” than psychotic disorders in terms of consequences of non-adherence [40].

Emerging LAIs for antidepressants remain relatively scarce, and most current development activity focuses on agents already used as atypical antipsychotics rather than truly on novel LAIs of SSRIs, SNRIs or MAOIs. For examples, the monthly Cariprazine depot LAI (#NCT07185815) and weekly Brexpiprazole LAI (#NCT05326347) SC depot injections have been studied primarily in schizophrenia, with early PK, safety studies and long-term phase 2 trials for MDD, bipolar disorder, and schizophrenia. Evidence relevant to LAI use in mood disorders also comes indirectly from bipolar and mixed affective populations [41]. Olanzapine and fluoxetine combination therapy has been evaluated in a 24-week double-blind randomized study for bipolar depression, with sustained remission in responders, while the long-acting olanzapine depot (Zyprexa Relprevv) remains primarily indicated for schizophrenia [42]. Similarly, aripiprazole lauroxil and related aripiprazole formulations have robust randomized controlled trial data as adjunctive oral therapy in MDD, with significant improvements in depression scores, whereas their LAI versions are licensed for schizophrenia and bipolar maintenance rather than for depression [43]. Similarly, long-acting risperidone and paliperidone depots, including monthly subcutaneous (SC) risperidone (Uzedy) [44] and monthly or three-monthly paliperidone palmitate (Invega Sustenna/Trinza), have accumulated extensive clinical and real-world data in schizophrenia, demonstrating delayed relapse, reduced hospitalization, and improved functional outcomes, and their indications now extend to bipolar maintenance, reinforcing their relevance to mood disorder trajectories even though they are not positioned as antidepressant LAIs [45,46].

From a patient-centered perspective, the most compelling rationale for LAI antidepressants comes from relapse and poor adherence outcomes in psychotic disorders rather than from direct antidepressant depot trials. Across multiple cohorts, LAI antipsychotics reduce relapse rates by roughly 20 to 30% compared with equivalent oral regimens and can extend relapse-free survival by months, largely by buffering against missed doses and abrupt discontinuation [47]. These benefits are mirrored by improvements in adherence, with LAIs often producing 5 to 20% absolute gains in treatment persistence and fewer unexpected discontinuations [48]. Given that non-adherence, pharmacokinetic instability, and early discontinuation contribute to recurrence in recurrent MDD, these antipsychotic-LAI data provide an indirect rationale for investigating LAIs in depression. However, they do not establish that antidepressant LAIs will produce comparable clinical benefits, which require direct evaluation in MDD populations. In the absence of late-phase studies for depression-specific LAI products, the existing LAI antipsychotic experience demonstrates that depot strategies can meaningfully alter long-term illness course when daily oral adherence is fragile. At the same time, the current development pipeline still lacks a fully realized, depression-specific monthly or quarterly LAI antidepressant in late phase 3, with much of the innovation concentrated in oral or non-injectable modalities such as novel receptor modulators and extended-release nasal or sublingual formulations.

## 2. Pharmacological and Clinical Rationale for LAI Antidepressants

Oral antidepressants such as selective serotonin reuptake inhibitors (SSRIs) and serotonin–noradrenaline reuptake inhibitors (SNRIs) have pharmacokinetic properties that make them vulnerable to the consequences of missed doses and premature discontinuation [49]. Drugs including Paroxetine and Venlafaxine, which have relatively short elimination half-lives among serotonergic antidepressants, are particularly prone to producing rapid declines in plasma concentrations when doses are missed or treatment is stopped, leading to a higher incidence and severity of discontinuation symptoms. These dynamics are compounded by interindividual variability in metabolism, drug–drug interactions, and comorbid medical conditions, all of which affect the peak–trough fluctuations under once-daily oral dosing. When dosing is inconsistent, patients are therefore exposed to repeated cycles of under- and overexposure, which may contribute to both suboptimal symptom control and adverse effects [50]. Such fluctuations result in withdrawal or discontinuation phenomena. SSRIs and SNRIs with short half-lives are especially associated with discontinuation syndromes and are characterized by symptoms such as insomnia, dizziness, sensory disturbances, and mood worsening when treatment is abruptly stopped or doses are missed [51]. Symptoms typically emerge within a few days of cessation and can last for days to weeks, and in some cases may be severe enough to interfere with daily functioning [52]. Importantly, these syndromes are often difficult to distinguish clinically from relapse of the underlying disorder, especially when they involve affective or anxiety symptoms, complicating decision-making around dose changes and discontinuation. Although gradual narrowing of doses can reduce the likelihood and severity of discontinuation reactions, implementing slow, individualized dosing in routine practice is challenging, and unplanned interruptions due to forgetfulness, access barriers, or intercurrent illness remain common [53].

Specific groups of patients are particularly susceptible to these limitations and may derive disproportionate benefit from LAI formulations. Poor adherence to continuation and maintenance of antidepressant treatment in recurrent depression is quite common and is associated with increased risk of recurrence, even among patients who have achieved remission [49]. Literature findings have shown that patients who discontinue antidepressants in early stages have a substantially higher risk of relapse or recurrence compared with those who maintain treatment as recommended [49]. Individuals with a history of recurrent major depressive episodes and those with cognitive impairment, comorbid substance use disorders, or unstable psychosocial circumstances may find consuming pills everyday particularly difficult to sustain over years [54]. Patients who are highly sensitive to discontinuation symptoms, including those treated with short-half-life agents such as paroxetine or venlafaxine, may also be at elevated risk of distressing withdrawal phenomena when doses are missed or treatment is interrupted [55].

LAI antidepressant formulations are conceptually designed to address these needs by providing sustained, predictable drug exposure over days, weeks or months with a single dose of injection. By improving the plasma concentration–time profile, LAI systems can minimize peak–trough variability, further reducing the dose-related side effects [39]. Sustained exposure could support continuity of antidepressant treatment, but improved adherence and reduced depressive relapse remain proposed benefits of antidepressant LAIs rather than established clinical outcomes. From a behavioral perspective, LAIs eliminate the cognitive and organizational demands of daily self-administration, transforming adherence into a sporadic, scheduled clinical routine that can be supported by appointment systems, reminder infrastructure, and multidisciplinary care pathways [40].

Potential clinical advantages of LAI extend beyond adherence; success from LAI antipsychotics suggests that LAI formulations can reduce relapse and hospitalization rates through improved adherence and by enabling regular, structured follow-up around injection visits. These visits create opportunities for systematic symptom monitoring, psychological education, assessment of side effects, and timely adjustment of psychosocial or pharmacological interventions [38]. LAIs may also lessen the stigma or internal conflict some patients experience around daily pill-taking by making treatment less visible in everyday routines. Although these putative benefits require empirical confirmation in the specific context of antidepressants, they are consistent with the broader literature on LAI in psychiatric care and provide a strong pharmacological and clinical rationale for advancing LAI antidepressant development [49]. However, supporting antidepressant maintenance treatment should be distinguished from evidence supporting LAI administration. In patients with MDD who achieved remission, continued antidepressant treatment was associated with lower relapse rates than discontinuation to placebo [56]. However, these findings do not establish that injectable delivery improves adherence or relapse prevention. Separately, studies comparing LAI and oral antipsychotics in schizophrenia have reported lower risks of hospitalization or relapse with LAIs [57]. These findings provide an indirect rationale for investigating antidepressant LAIs, but comparable adherence and relapse benefits in MDD require direct clinical evaluation.

## 3. Technological Platforms for LAI Antidepressants

A range of injectable sustained-release platforms originally developed for peptides, antipsychotics, and other chronic therapies are now being widely explored. These systems differ in mechanisms of depot formation, release kinetics, dose-loading capacity, and scalability, all of which are critical for relatively high doses of small molecules such as antidepressants. In the below sections, each of the LAI approaches, including polymeric microspheres, polymeric nanoparticles, in situ-forming depots (ISFDs), lipid-based depots, and hybrid/emerging systems, is discussed with their key contributions in modulating the long-term release, preserving drug stability, and ensuring safe excipients and injection volumes.

### 3.1. Polymer-Based LAI Systems

**Figure 4:**
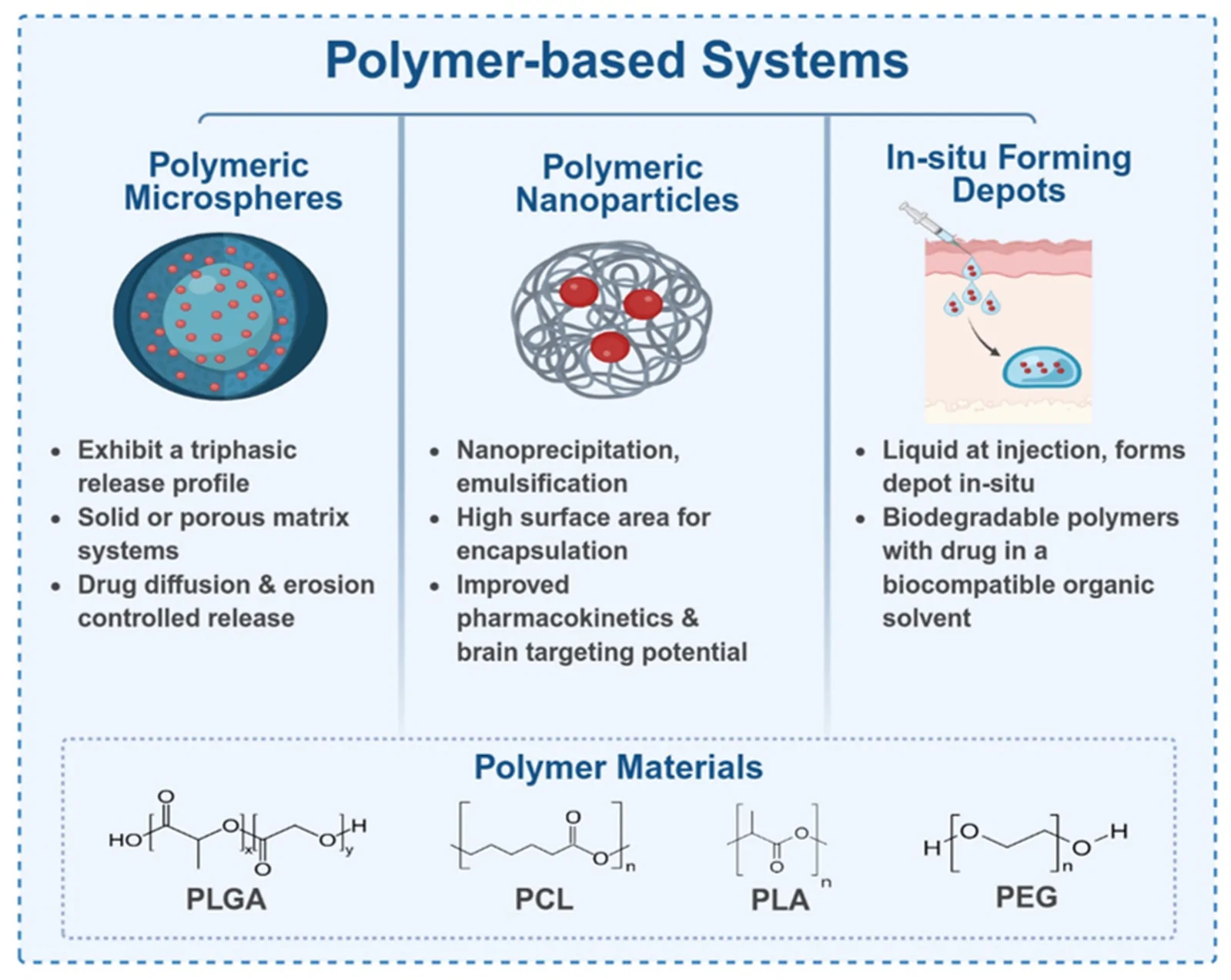
Polymer-based LAI delivery systems: microspheres, nanoparticles, and ISFDs [created using Biorender; Garg. S (2026) https://BioRender.com/w4kkrfu].

Polymer-based LAI systems constitute one of the most versatile and widely explored platforms for sustained drug delivery. These platforms can be designed as polymeric microspheres, nanoparticles or in situ-forming depot systems (Figure 4) and can modulate the release kinetics, improve the drug stability, and extend therapeutic exposure. A wide range of polymers including synthetic, semi-synthetic, and natural types have been investigated for LAI formulations, each offering distinct physicochemical properties, degradation behavior and biocompatibility profiles [58]. In the context of CNS-active agents, this platform technology is particularly attractive in addressing non-adherence and pharmacokinetic variability and improving drug stability [59]. A broad range of polymers has been explored for LAI systems, including polyesters such as poly(lactic acid) (PLA), poly(glycolic acid) (PGA), polycaprolactone (PCL), and their copolymers (such as PLGA), as well as polycarbonate (PC)- and polyethylene glycol (PEG)-based copolymers and natural polymers such as chitosan, alginate, gelatin and hyaluronic acid [60].

#### 3.1.1. Polymeric Microspheres

Polymeric microspheres represent one of the most established biodegradable depot platforms for LAI delivery, enabling sustained release of small-molecule biologics over weeks to months through a combination of diffusion and polymer degradation [61]. Typically prepared from biodegradable polymers such as PLA and PLGA, these systems exhibit a characteristic triphasic release pattern: an initial burst phase caused by a drug associated with or near the particle surface, a prolonged diffusion-controlled phase through the hydrated polymer matrix, and a final accelerated phase associated with bulk erosion and mass loss of the polymer [62]. Their release behavior can be tuned by adjusting polymer molecular weight, hydrophilicity, architecture, and related formulation parameters, which influence water uptake, autocatalytic acidification, and erosion kinetics. Microspheres are commonly manufactured by emulsion-solvent evaporation, spray drying, or phase separation, and after administration they swell as water penetrates the particles, allowing drug diffusion and gradual matrix erosion to drive release [63].

Despite these advantages, formulating antidepressants into microspheres presents several challenges. For small hydrophilic or amphiphilic molecules, encapsulation efficiency is often limited because a substantial fraction of drug partitions into the external aqueous phase during manufacturing, necessitating strategies such as pH adjustment, ion pairing, or polymer optimization to improve loading [62]. Burst release also remains a major concern and can be reduced by controlling particle size distribution, minimizing surface-associated drugs, using higher-molecular-weight polymers, or applying surface coatings. In addition, both the drug and polymer must remain stable during exposure to organic solvents, shear stress, and the acidic microenvironment generated during polymer degradation, which can promote hydrolysis or oxidation [64]. The final microsphere suspension must also remain physically stable and injectable through 22–25 G needles, requiring appropriate suspending vehicles and surfactants to prevent aggregation while maintaining acceptable viscosity [65]. Nevertheless, microspheres remain attractive for antidepressant LAIs because PLA/PLGA-based systems have extensive clinical precedent and well-characterized degradation behavior, and they can be engineered for depot durations ranging from weeks to several months, with applicability to both intramuscular (IM) and subcutaneous (SC) administration [66].

Studies from the literature further suggest how formulation engineering can improve microsphere performance for poorly soluble drugs. Nanaki et al. used mesoporous cellular foam (MCF) silica as a solubility-enhancing agent. Before microsphere encapsulation of paliperidone, the drug was converted to an amorphous form, showing improved dissolution, and when further encapsulated into PLA and PLGA 75:25 microspheres it achieved drug loading of up to 15% with release over 10–15 days, demonstrating a meaningful gain in both solubility and sustained-release behavior [67]. In a related study, the same group improved the loading of naltrexone in poly(L-lactic acid)-block-poly(pentadecalactone) (PLLA-b-PLLAd) microspheres, raising encapsulation to 55 to 62% and enabling 82 to 90% drug release over 7 days through diffusion-controlled release after conversion to an amorphous state [68]. Together, these findings show that combining solubility enhancement with polymeric microsphere matrices can be highly effective for difficult compounds, and the same principles may be relevant to antidepressants with low aqueous solubility such as paroxetine, as well as drugs with very different solubility profiles such as bupropion.

The clinical relevance of this platform is further supported by risperidone microspheres, where Son et al. engineered PLGA microspheres to achieve continuous release for approximately 40 to 50 days, eliminate the lag phase, and maintain more stable plasma concentrations despite the drug’s short half-life [69]. These examples highlight the strengths of microsphere systems as they can improve dosing convenience, stabilize exposure, and extend drug action without relying on daily oral administration. At the same time, important limitations remain for antidepressant applications, including relatively high maintenance dose requirements, constrained drug-loading capacity, low encapsulation efficiency for some SSRIs, acid-mediated degradation of sensitive compounds, injection-site irritation, and manufacturing and scale-up challenges associated with strict particle-size control [70,71,72,73]. Overall, polymeric microspheres remain one of the most mature and clinically validated LAI strategies, but their suitability for antidepressants will depend on corresponding formulation design to drug dose, solubility, stability, and injectability requirements.

#### 3.1.2. Polymeric Nanoparticles

Polymeric nanoparticles typically range from 50 to 300 nm and represent the smallest class of biodegradable particulate systems used for sustained drug delivery. Since the particle size is small, it offers larger specific surface area and rapid hydration; this leads to gradual diffusion of drug molecules through the polymer matrix while the polymer undergoes slow relaxation and degradation, enabling sustained release over hours to weeks [74]. Following IM or SC administration, the polymeric nanoparticles can form localized depots that drain slowly into systemic circulation, whereas intravenous administration allows them to function as circulating reservoirs that modulate biodistribution and release rate [75]. For antidepressants, parenteral administration is conceptually attractive because depot-like delivery can extend dosing intervals and improve adherence, while systemic nanoparticle approaches may also be useful when CNS exposure needs to be enhanced [76].

The performance of polymeric nanoparticles depends strongly on the fabrication method used. Nanoprecipitation is a widely used method as it is simple and scalable and avoids high shear, producing particles by solvent displacement from a water-miscible organic phase, although encapsulation can be lower for hydrophilic or ionized drugs [77]. In contrast, emulsion-solvent evaporation more closely resembles microsphere preparation and can improve drug loading, but both methods influence particle size, polydispersity, surface chemistry, and drug distribution within the matrix, all of which directly affect release rate and stability. Because of their high surface-area-to-volume ratio, polymeric nanoparticles generally hydrate faster and can provide more predictable release than larger particulate systems, making them appealing for antidepressants where both rapid onset and sustained maintenance are desirable [78].

Recent studies using PEGylation and ligand conjugation can reduce opsonization and clearance, prolong circulation time, and in some cases improve blood–brain barrier penetration, thereby increasing the brain-to-plasma ratio for CNS-active drugs [79]. A representative example is haloperidol-loaded PLGA nanoparticles prepared by Budhian et al., which achieved around 20% drug loading, converted haloperidol from a crystalline to an amorphous state within the polymer matrix, and achieved sustained release for 2 to 3 weeks while improving bioavailability and maintaining therapeutic plasma levels [80]. Similarly, Kumar et al. [81] reported PEGylated nano-assisted delivery systems with 25 to 35% encapsulation efficiency, prolonged circulation, enhanced brain targeting, and sustained release over 30 days, with zero-order kinetics and a 3–4-fold increase in brain-to-plasma ratio, demonstrating the translational potential of such systems for antipsychotics and, by extension, lipophilic antidepressants. Together, these findings show that polymeric nanoparticles can function as effective long-acting CNS delivery systems when amorphization, controlled degradation, and surface engineering are combined [82].

Compared with microsphere-based strategies, nanoparticle systems offer a distinct performance profile. Their smaller size and greater surface area can favor faster hydration, more uniform drug distribution, and enhanced tissue penetration, which may be advantageous when a quicker onset of action is desired while still maintaining sustained exposure. For antidepressants such as paroxetine, nanoparticle platforms may be especially useful when paired with PEGylation for BBB-related delivery or with block copolymer matrices to improve loading, analogous to the loading gains reported in block-copolymer microsphere systems [83,84]. However, important translational limitations remain, including lower drug capacity for some drugs, complex physicochemical characterization, narrow size-distribution requirements, sterilization sensitivity, and evolving regulatory expectations for nanomedicines [75]. As a result, polymeric nanoparticles are capable but not yet the most straightforward commercial LAI option for high-dose antidepressants, though they remain highly relevant for compounds where brain delivery and controlled exposure are central design goals [74,85]. To conclude, the antidepressant-specific nanocarrier studies should be distinguished from evidence for systemic LAI depots. The experimental findings using alternative administration routes or short observation periods do not, by themselves, establish sustained exposure after IM or SC injection.

#### 3.1.3. In Situ-Forming Depots (ISFDs)

Polymeric in situ-forming depots (ISFDs) represent a well-established class of LAI systems in which a low-viscosity formulation is administered as a liquid and then transforms in situ into a solid or semi-solid depot after injection (commercially available ISFIs are listed in Table 3). These systems typically contain a biodegradable polymer dissolved or dispersed in a biocompatible organic solvent such as dimethyl sulfoxide or N-methyl-2-pyrrolidone [86]. Once injected into the aqueous environment of muscle or SC tissue, rapid solvent–physiological fluid exchange at the formulation interface drives polymer phase inversion, causing it to lose its solubility and precipitate into a coherent polymer–drug matrix at the injection site [87]. In the early post-injection phase, a drug located in solvent-rich regions or near the depot surface can diffuse more rapidly into surrounding tissue, contributing to an initial burst, whereas later release becomes governed by diffusion through the hydrated polymer network and, over time, by polymer erosion and mass loss, together sustaining drug release over days to weeks or even months [88,89].

Designing an ISFD therefore depends on carefully balancing polymer properties, solvent composition, and drug-excipient interactions. Higher polymer concentration and molecular weight generally increase solution viscosity and depot firmness, slowing diffusion and degradation but also making injection more difficult [88,89]. The choice and proportion of solvent determine the rate of phase inversion and the eventual porosity or density of the formed matrix. A fast solvent exchange can generate a highly porous depot and a larger initial burst, whereas slower phase inversion can smooth early release but may affect depot integrity [90]. In addition, drug solubility and partitioning between the polymer–solvent phase and the surrounding aqueous phase strongly determine how much of the dose is lost early versus retained for long-term release. Hybrid architectures that incorporate secondary structures such as dispersed hydrogels or microparticles can further dampen burst release and refine release kinetics for both water-soluble and lipophilic small molecules [91].

ISFDs offer several reasonable advantages for LAI development. They can be formulated as homogeneous solutions or low-viscosity suspensions, supporting relatively high drug loading and dose adjustment by changing injection volume rather than redesigning the formulation. The absence of pre-formed particles also simplifies manufacturing by eliminating steps such as particle formation, drying, and milling, and allows the depot to conform to local tissue geometry, which may improve distribution [92]. However, these systems depend on organic solvents that must meet stringent safety criteria and may cause local pain or irritation during phase inversion. If solvent exchange is too rapid or the drug is highly soluble in the solvent, substantial initial burst release can occur and may produce supra-therapeutic early exposure [91]. Depot formation and long-term performance are also sensitive to injection technique, anatomical site, and local tissue characteristics, which can lead to variability between patients and even between injections in the same patient [87,93]. For antidepressant delivery, these systems are particularly attractive when high total doses are needed but the drug remains sufficiently soluble and stable in the selected polymer–solvent matrix. Overall, ISFDs occupy a feasible strategy for long-acting antidepressant therapy, especially where monthly dosing, reduced dosing burden, and controlled release are clinically important.

Recent studies further show how ISFDs can be tuned for CNS drugs and other challenging molecules. Shiadeh et al. developed a PLGA-PEG-PLGA triblock copolymer-based ISFD for risperidone delivery, which formed a gel depot after injection and achieved one-month sustained release in rabbits with improved pharmacokinetics compared with risperidone microspheres, including the absence of an initial lag phase and more consistent plasma levels [94]. Similarly, a sucrose acetate isobutyrate-based ISFD for risperidone provided sustained release for 30 days with minimal burst (<20% in the first 24 h) and stable plasma concentrations, while avoiding the processing steps required for microspheres and supporting higher drug loading through a non-swelling matrix [95]. In another study, PLGA-based ISFDs were adapted for naltrexone by tuning solvent blends to regulate precipitation kinetics and depot formation, producing 80 to 90% release over 30 days with burst levels below 15%. This depot geometry also improved retention at the injection site and aligned with monthly LAI requirements [86]. Abulateefeh et al. showed that PLA-based ISFDs could be tuned by end-capping and polymer concentration, achieving release for up to 60 days, with ester end groups producing smoother profiles and less burst than acid end groups [96]. Likewise, PLGA-based ISFDs optimized by lactic-to-glycolic acid ratio sustained paclitaxel release for up to 21 days and improved antitumor efficacy in vivo compared with Taxol^®^, while clonazepam-loaded PLGA-based ISFDs using NMP achieved about 90% release over 28 days in vitro and stable plasma exposure in rabbits, with drug amorphization improving release of the solubility-limited cargo [97]. Taken together, these studies establish ISFDs as a flexible LAI platform with sustained release ranging from days to months, high design versatility, and strong translational relevance for CNS drugs and suggest that, with careful matching of polymer, solvent, and drug properties [94], ISFDs could be a highly feasible approach for antidepressant LAIs, particularly for patients who would benefit from monthly regimens and improved adherence.

**Table 3:** Commercially available polymer-based ISFD LAIs.

Product Name	Active Ingredient	Indication	Main Polymer System	Solvent System	Duration	Key Features and Significance	Ref.
Eligard^®^	Leuprolide acetate	Prostate cancer (GnRH agonist)	PLGA	N-methyl-2-pyrrolidone (NMP)	1–6 months	First major ISFD success; monthly to 6-month administration based on dosing; proves systemic peptide delivery via simple injection	[98]
Camcevi^®^	Leuprolide acetate	Prostate cancer (GnRH agonist)	PLGA	N-methyl-2-pyrrolidone (NMP)	6 months	6-month injection, proves systemic peptide delivery via simple injection	[99]
Atridox^®^	Doxycycline hyclate	Periodontal disease (local)	PLA/PLGA biodegradable	N-methyl-2-pyrrolidone (NMP)	~21 days	Pioneering local ISFD; forms depot in periodontal pocket, validates site-specific delivery	[100]
Sublimaze^®^	Fentanyl citrate	Pain management (epidural)	Biodegradable polymer	Organic solvent	~48 h	One of the earliest FDA-approved ISFDs, demonstrates short-to-medium duration	[101]
Perseris^®^	Risperidone	Schizophrenia (antipsychotic)	PLGA	NMP-based	Monthly	Psychiatric LAI via ISFD, reduces burst and improves adherence in CNS disorders	[102]

### 3.2. Lipid-Based Depots

**Figure 5:**
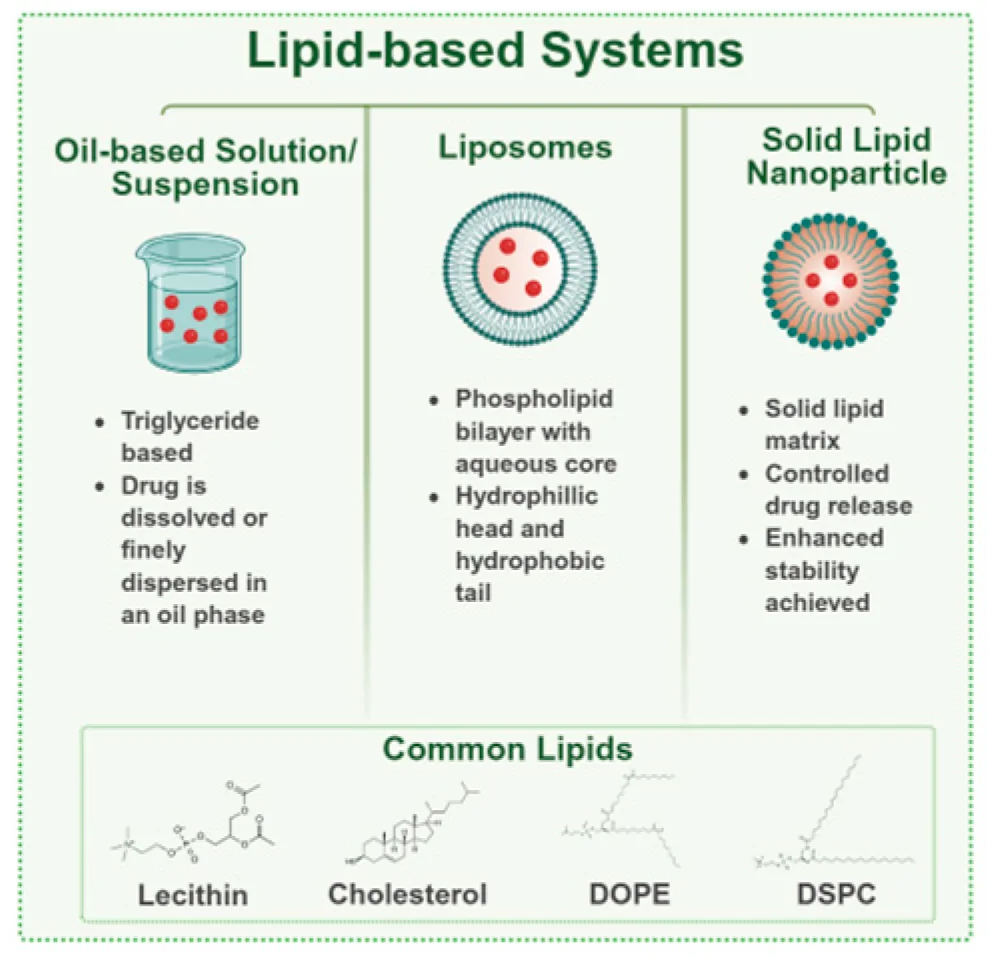
Lipid-based LAI delivery systems: oil-based suspensions/solutions, liposomes, and solid-lipid nanoparticles [created using Biorender; Garg. S (2026) https://BioRender.com/w4kkrfu].

Lipid-based depots comprise a diverse group of LAI systems in which drug release is controlled primarily by lipid excipients rather than biodegradable polymer matrices [103]. The most established approach is the use of oil solutions or suspensions, in which a lipophilic drug or ester prodrug is dissolved or dispersed in a biocompatible triglyceride-based vehicle. Following IM or SC administration, drug release occurs through gradual partitioning from the oil depot into surrounding aqueous tissue fluids, together with slow clearance or resorption of the vehicle [104]. Other lipid-based approaches include liposomes, particularly multivesicular liposomal systems, which encapsulate hydrophilic drugs in aqueous compartments or associate lipophilic drugs with phospholipid bilayers, resulting in drug release through diffusion, vesicle disruption, and cellular uptake [105]. More recently, solid lipid nanoparticles (SLNs), nanostructured lipid carriers (NLCs), liquid-crystalline depots, and lipid-based ISFDs have been investigated to provide additional control over release kinetics, tissue distribution, and tolerability. However, unlike established oil depots and some liquid-crystalline or multivesicular-liposomal products, lipid nanoparticles remain mainly preclinical or early translational platforms for parenteral sustained delivery (Figure 5) [105].

For antidepressant applications, formulation design must account for the drug’s lipid solubility and partitioning between lipid and aqueous environments, since these characteristics strongly influence loading, release rate, and depot persistence. In oil depots, the choice of triglyceride vehicle, drug concentration, viscosity modifier, and any co-solvent influences injectability, depot spreading, resorption kinetics, and local tolerability [106]. In liposomal and lipid-nanoparticle systems, drug stability may be enhanced by incorporation within lipid bilayers or solid/semi-solid matrices. However, these colloidal formulations also require strict control of size distribution, surface charge, lipid composition, aggregation, fusion, and phase separation to ensure reproducible release and acceptable shelf-life. These physicochemical factors can further affect circulation, tissue uptake, and, where relevant, CNS distribution, although enhanced brain delivery should be considered formulation-specific rather than assumed [107].

Lipid depots offer several advantages, including the use of generally biocompatible excipients with established parenteral precedent and, for oil solutions, relatively simple and scalable manufacture. They can be especially attractive for highly lipophilic small molecules or ester prodrugs, which may be formulated at high concentrations and administered at intervals of several weeks without exposing the drug to acidic polyester degradation products [108]. Nonetheless, oil depots may cause injection-site pain, nodules, and variable absorption, whereas liposomal and lipid-nanoparticle dispersions can have limited loading for hydrophilic drugs and are more vulnerable to physical instability during storage and handling. Consequently, lipid-based systems may be most suitable for lipophilic antidepressants when an intermediate duration such as two to four weeks is clinically adequate, or when avoidance of polymer-associated acidity is a priority [109].

Selected marketed lipid-based LAIs (as mentioned in Table 4) demonstrate that this approach is clinically feasible across psychiatric, endocrine, analgesic, and addiction-treatment indications. Classical oil-based antipsychotic depots, including haloperidol decanoate and fluphenazine decanoate, use lipophilic ester prodrugs dissolved in sesame oil and are typically administered by the IM route at intervals of approximately two to four weeks. Their clinical utility derives from sustained plasma exposure and reduced reliance on daily oral therapy, which can improve adherence in schizophrenia [110]. Emerging lipid-depot precedents include liquid-crystalline systems and multivesicular liposomes, demonstrating that lipid technologies can extend beyond conventional oil solutions to provide structured depots with more tunable release profiles [111]. Collectively, these marketed products establish oil-based depots as clinically validated lipid LAIs and provide a precedent for developing intermediate-duration formulations of suitably lipophilic antidepressants.

Collectively, these marketed non-antidepressant products establish that oil-based depots can provide clinically useful long-acting delivery for selected lipophilic drugs. They provide indirect formulation precedent for evaluating intermediate-duration antidepressant formulations, rather than direct evidence of antidepressant-LAI safety or efficacy [114]. Especially in conditions like schizophrenia, these depot formulations can improve adherence and maintain more consistent drug exposure. Clinical evidence has associated their use with lower relapse and hospitalization rates than oral treatment in appropriate patient populations [115]. A comprehensive review by Wilkinson et al. of marketed and emerging lipid-based IM LAIs further identified oil-based antipsychotic and endocrine depots as established technologies that can provide durable therapeutic exposure without requiring biodegradable polymer matrices [103]. These precedents provide adoptable guidance for antidepressant LAI design, particularly in matching the lipid platform to the required dose, dosing interval, injection volume, and desired systemic or CNS distribution profile [116].

### 3.3. Hybrid and Emerging Systems (Stimulus-Responsive, Injectable Hydrogels)

**Figure 6:**
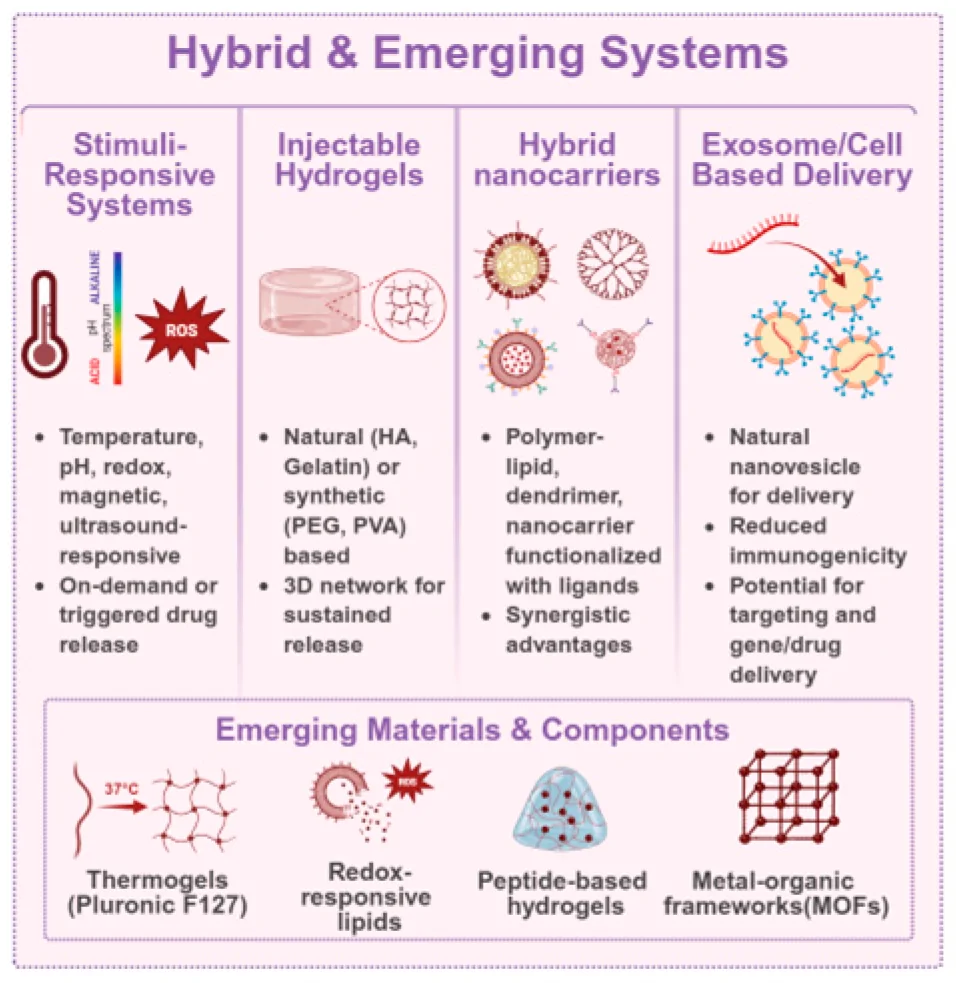
Hybrid or emerging platforms for LAI drug delivery systems [created using Biorender; Garg. S (2026) https://BioRender.com/w4kkrfu].

Hybrid and emerging depot systems (Figure 6) represent a newer generation of LAI technologies designed to address limitations associated with conventional polymeric and lipid depots. Rather than relying on a single material class, these platforms combine polymeric and lipid components or incorporate stimulus-responsive features that permit more precise control of depot formation and drug release [117]. Injectable hydrogels are a prominent example that are administered as low-viscosity liquids and subsequently form soft, hydrated networks in situ in response to physiological temperature, ionic strength, pH, or other triggers [118]. Other approaches include polymer–lipid hybrid particles and bulk depots, as well as pH-, enzyme-, redox-, or light-responsive systems whose release behavior changes with local environmental conditions. Drug release is commonly controlled by diffusion through the hydrated matrix, matrix swelling, network erosion or degradation, and, in certain responsive systems, reversible changes in mesh size or intermolecular interactions triggered by the local microenvironment [119].

Despite their design and versatility, hybrid and stimulus-responsive depot systems remain substantially less mature than established microsphere, ISFD, and oil-based LAI technologies. Most reported responsive hydrogels are supported by in vitro experiments or short-duration preclinical studies, often using oncology or local delivery models rather than repeated systemic administration for chronic psychiatric treatment. Their apparent advantages, including reduced burst release, on-demand release, and multi-drug loading, should therefore be interpreted as proof-of-concept potential rather than demonstrated clinical benefit [120,121]. Translation of this technology will require evidence that supports the crosslinking chemistry, trigger mechanism, and safety of degradation products during repeated administration, and that their release remains predictable under physiologically practical variation in tissue temperature, pH, ionic strength, inflammation, and enzymatic activity [122]. Most evidence for these systems is derived from non-antidepressant and frequently non-psychiatric models; therefore, their relevance to systemic antidepressant LAIs remains conceptual until validation using antidepressants, intended injection routes, chronic dosing regimens, and clinically relevant endpoints.

However, successful translation of these systems depends on several practical formulation requirements. The formulation must remain syringeable through a clinically acceptable needle while forming a reproducible depot under IM or SC conditions. The drug must also retain chemical and physical stability during gelation and throughout its residence in the matrix, despite potential exposure to altered temperature, pH, water activity, or crosslinking conditions [123]. For stimulus-responsive systems, the trigger must be both sufficiently reliable and clinically feasible. Body temperature is particularly attractive because it is predictable and universally available after injection, whereas pH-, enzyme-, or externally triggered systems may be more relevant to localized or disease-specific applications than to conventional systemic LAIs [124]. When these requirements are met, hybrid networks can be tailored to reduce burst release, approach sustained or near-zero-order release profiles and potentially co-deliver multiple agents in a single injection. However, their complex architecture also complicates the scale-up, sterilization, quality control, and batch-to-batch reproducibility [125].

A particularly relevant example for long-acting systemic delivery was reported by Dutta et al., where the study developed injectable thermoresponsive hydrogels based on PLGA-PEG-PLGA triblock copolymers for peptide and protein delivery. The formulations underwent a temperature-induced sol-to-gel transition near body temperature, forming ISFDs that released active cargos for 4 to 35 days. Release duration was adjusted through polymer concentration, polymer hydrophilic–hydrophobic balance, and the size and solubility of the encapsulated peptide or protein. Importantly, the study showed that thermoresponsive hydrogels can sustain release without organic solvents and preserve protein conformation during encapsulation and release, making this platform especially relevant to long-acting peptide, protein, or biologic therapies [126]. Hybrid hydrogels can also incorporate local microenvironment-responsive release mechanisms. For example, an injectable gelatin–PEG/Laponite hybrid hydrogel was developed as a self-healing, pH-responsive depot for doxorubicin delivery in local cancer therapy. The formulation contained doxorubicin-loaded Laponite nanodiscs dispersed within a chemically crosslinked gelatin–PEG network and underwent gelation in approximately 80 s following injection. It exhibited pH-dependent long-term release, with faster doxorubicin liberation under acidic, tumor-relevant conditions, together with good biocompatibility and anticancer activity in cell-based studies [127]. Although this system was designed for oncology rather than systemic psychiatric therapy, it demonstrates how a hybrid network can integrate injectability, mechanical recovery, nanoparticle incorporation, and environment-responsive release within one depot.

External stimuli offer an additional means of controlling drug release from long-acting hydrogel depots. Xu et al. developed an injectable NIR/pH-responsive hydrogel containing gold nanorods and an acid-labile doxorubicin prodrug; acidic conditions and NIR-induced photothermal heating accelerated drug release for chemo-photothermal therapy [128]. Similarly, an injectable thermoresponsive mPEG-PLGA-BOX hydrogel provided sustained doxorubicin release for approximately one week, while low-intensity ultrasound reversibly increased release by about 70-fold in vitro and 10-fold in vivo [129]. Magnetic hydrogels containing superparamagnetic iron oxide nanoparticles have also enabled pulsatile release under an alternating magnetic field through local heating and reversible network changes [120]. Together, these systems demonstrate how light, ultrasound, or magnetic fields can provide on-demand release control; although largely preclinical and oncology-focused, ultrasound and magnetic triggering may be more easier approaches than NIR for future IM or SC antidepressant LAIs [121].

Overall, hybrid and stimulus-responsive hydrogels offer long-term opportunities to refine depot formation, reduce burst release, and potentially enable combination or externally modulated therapy. However, their clinical translation remains in early stages and should not be considered equivalent to that of established microspheres, ISFDs or lipid depots [130]. Near-term development of antidepressant LAIs is most likely to rely on established depot technologies with existing parenteral excipient, manufacturing, and regulatory precedent. Among the emerging systems, biodegradable thermoresponsive hydrogels may be comparatively more feasible delivery systems as body temperature is a predictable physiological trigger and avoids the need for external devices [125,131]. Nevertheless, hybrid systems still require demonstration of long-term biocompatibility, reproducible gelation and release, sterilization compatibility, scalable manufacturing, and acceptable local tissue tolerance. In contrast, pH-, enzyme-, NIR-, ultrasound-, and magnetic-responsive systems should currently be regarded as exploratory technologies. Their translation is limited by uncertainty regarding trigger reliability in vivo, tissue penetration or device requirements, crosslinker and nanomaterial safety, batch-to-batch reproducibility, and absence of demonstrated clinical advantage over simpler depot technologies [127,128,129].

## 4. Cross-Platform Comparative Analysis

**Figure 7:**
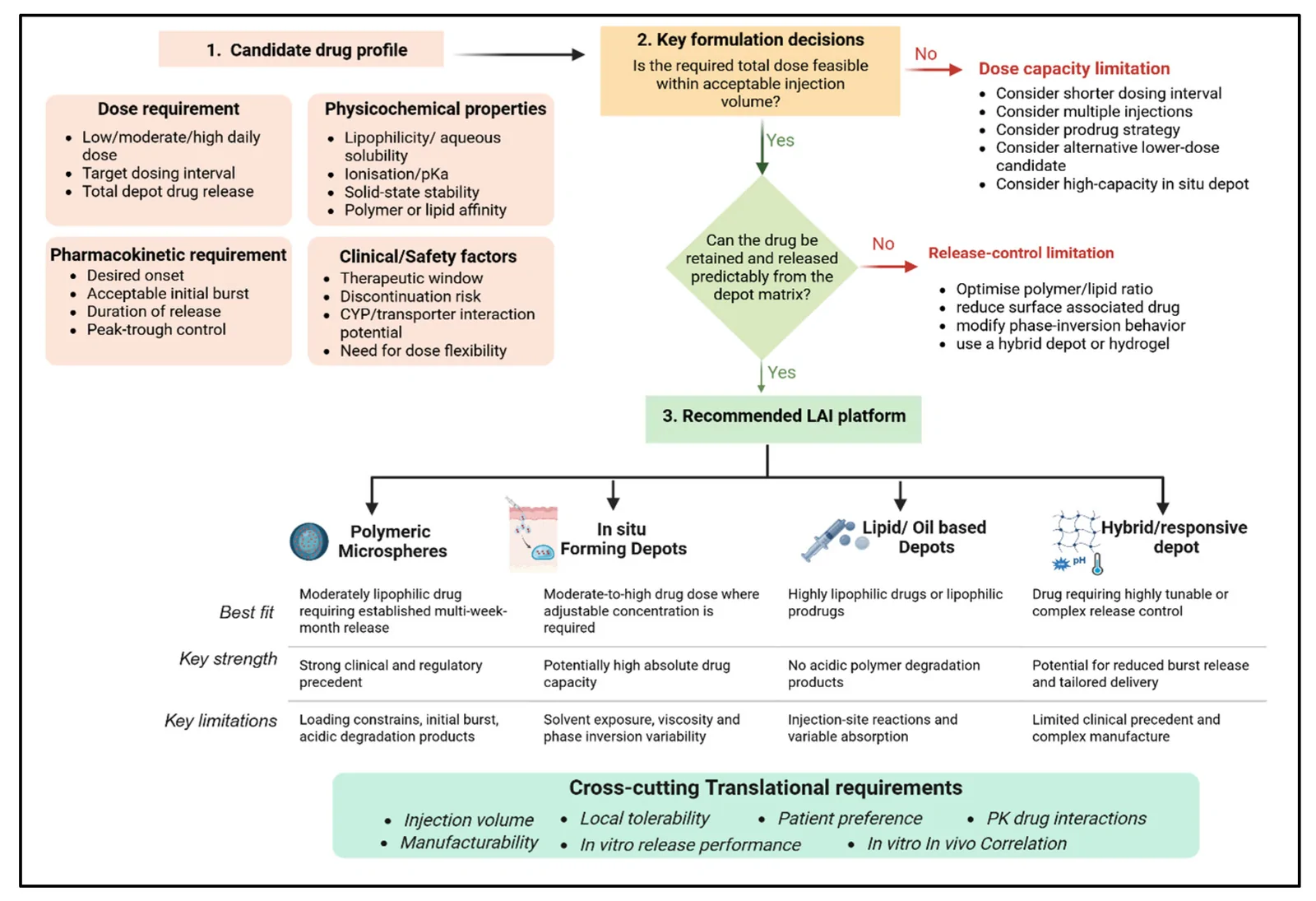
Conceptual framework for selecting LAI antidepressant platforms [created using Biorender; Garg. S (2026) https://BioRender.com/w4kkrfu].

Long-acting platforms differ in depot-formation mechanisms, drug loading capacities, manufacturing feasibility, and regulatory aspects. These differences are especially critical for relatively high-dose small molecules such as antidepressants. In general, these principles can be drawn from established experience with PLGA/PLA microspheres, in situ-forming depots, and lipid-based injectables in other drug classes, as well as from broader overviews of LAI technologies. They can be used to evaluate the crucial parameters that can be used in designing delivery technologies based on suitability to different antidepressants. To facilitate a deep understanding of release mechanisms, drug loading, injection feasibility, manufacturing and regulatory feasibility are evaluated based on existing literature to provide a cross-comparison among the delivery technologies and create a basis for deciding the best approach to start with (as described in Figure 7).

### 4.1. Suitability for Specific Antidepressant Classes

Considering the critical factors listed above, no single LAI technology will be suitable for every antidepressant. Platform selection should be based on each molecule’s dose requirement, lipophilicity, ionization, aqueous and lipid solubility, pharmacokinetic half-life, therapeutic window, and clinical consequences of delayed release or abrupt loss of exposure. Moderately to highly lipophilic antidepressants including fluoxetine, sertraline, duloxetine, and mirtazapine may be suitable candidates for polymeric microspheres or ISFDs provided that sufficient drug loading and control of initial burst release can be achieved. Fluoxetine, sertraline, and duloxetine are weak lipophilic bases, which can favor partitioning into hydrophobic polymer matrices. However, their relatively high oral maintenance doses mean that feasibility must ultimately be confirmed by achievable loading and acceptable injection volume.

Among the proposed candidates, fluoxetine has a relatively long elimination half-life and is a lipophilic weak base; this property may mitigate the sudden troughs in concentration but does not resolve its total dose-loading requirement [132]. Sertraline is highly lipophilic and may have favorable affinity for hydrophobic polymer or lipid matrices. However, its usual maintenance dose can be substantial, making achievable drug loading and injection volume critical [133]. Duloxetine is a lipophilic weak base but has a shorter half-life than fluoxetine and has potential CYP-mediated interaction. Therefore, release reproducibility, end-of-interval exposure, and drug-interaction management would be important design considerations [134]. Mirtazapine is a lipophilic basic compound with moderate oral dose requirements and may warrant exploration in polymeric or lipid-based depots. However, its suitability will depend on its solubility in the selected matrix, physical stability, attainable loading, and the intended dosing interval [135]. These proposed drug–platform relationships are hypotheses based on physicochemical and pharmacokinetic properties, rather than experimentally established LAI suitability, where no reported formulation study is available. Achievable loading, drug–excipient compatibility, release performance, and injection volume must therefore be determined for each drug–platform combination.

For agents associated with clinically relevant discontinuation symptoms, sustained and reproducible release is particularly important. Venlafaxine presents the clearest example, as abrupt cessation is associated with frequent and potentially severe withdrawal symptoms, and citalopram, escitalopram, and sertraline are generally regarded as having a more moderate risk [136]. LAI delivery may reduce the discontinuation symptoms caused by missed daily oral doses by maintaining more consistent systemic exposure. However, this benefit depends on the reliable depot performance through the dosing interval. If drug release falls below the therapeutic range because of premature depot depletion, an extended lag phase, variable absorption, delayed reinjection, or an interaction that increases drug clearance, the resulting decline in plasma concentration may still trigger discontinuation symptoms. This risk may be particularly be relevant for short-half-life antidepressants, such as venlafaxine and paroxetine, for which rapid reductions in exposure are associated with frequent withdrawal phenomena [52]. Therefore, LAI formulations intended for such drugs should demonstrate controlled terminal release, adequate end-of-interval trough concentrations, and a clinically justified missed-dose or oral supplementation strategy. A prolonged duration of nominal drug release alone should not be considered sufficient evidence of protection against discontinuation symptoms [137,138].

Microspheres with low surface-associated drug, carefully optimized ISFDs, or hydrogel systems could theoretically reduce peak–trough fluctuation and lessen the risk of an abrupt fall in exposure near the end of the dosing interval. However, an LAI does not eliminate discontinuation risk if dosing is delayed, treatment is stopped, or the depot cannot be removed [139]. Highly lipophilic agents, including many tricyclic antidepressants (TCAs) such as amitriptyline, nortriptyline, imipramine, and clomipramine, may be more compatible with oil-based or structured lipid depots because these vehicles can dissolve or suspend lipophilic drugs without producing acidic polymer degradation products. Mirtazapine and trazodone may also warrant evaluation in lipid-based systems, although suitability depends on drug solubility, dose, stability, and the intended dosing interval rather than lipophilicity alone. However, the proposed suitability depends on measured solubility in the selected vehicle, attainable concentration, stability, release behavior, and local tolerability rather than lipophilicity alone [107].

Nanoparticle and liposomal systems have a more specialized role. Their size and surface properties may alter tissue distribution and cellular uptake, but they should not be assumed to enhance central nervous system penetration after systemic administration as crossing the blood–brain barrier remains a major challenge. Their most plausible near-term application may be as components of hybrid depot systems that modulate release or local drug distribution, rather than as standalone high-capacity LAIs for conventional antidepressant doses [140]. Hybrid and stimulus-responsive depots may eventually support personalized dosing, combination therapy, or externally modulated release. However, their additional formulation, manufacturing, safety, and regulatory complexity mean that conventional microspheres, ISFDs, and simple lipid systems are more reliable first-generation platforms [137]. Existing experience with LAI technologies provides a useful development foundation, but antidepressant-specific pharmacokinetic targets, tolerability requirements, and discontinuation risks will still need to be established experimentally.

### 4.2. Drug-Release Mechanisms and Achievable Duration

Designing LAIs for antidepressants ultimately requires matching each drug to a platform whose intrinsic release mechanism aligns with its pharmacology and the clinical goals of maintenance therapy. Polymeric microspheres based on PLGA, PLA, or PCL generally exhibit multiphasic release, including an initial diffusion-driven burst from the surface-associated drug, a slower phase governed by water ingress and diffusion through the hydrated matrix, and a later phase associated with polymer erosion and mass loss [62]. This profile is attractive for long-term maintenance, but only when the initial burst is tightly controlled, because chronic antidepressant treatment requires stable exposure and abrupt concentration changes may increase dose-related adverse effects or contribute to discontinuation symptoms. Accordingly, microsphere formulations should be engineered to minimize early release and maintain drug concentrations within the therapeutic range without pronounced peak–trough fluctuations, making burst control a central design objective for antidepressant depots [141].

In contrast, polymeric nanoparticles generally have a higher surface-area-to-volume ratio and shorter diffusion pathways, which can favor faster hydration and more rapid drug release than larger particulate systems [142]. Consequently, nanoparticles alone may be less suitable for achieving monthly or multi-month release of standard-dose SSRIs or SNRIs, unless their release is moderated by incorporation into a secondary depot, such as an injectable hydrogel or an in situ-forming matrix [143]. ISFDs occupy an intermediate position as they undergo solvent exchange after injection to form a solid or semi-solid polymer matrix, with early release dominated by drugs in the solvent-rich phase and later release governed by diffusion and polymer degradation [86]. Like microspheres, these systems require careful suppression of early burst release. However, their ability to accommodate higher drug loads and adjust doses through injection volume may make them particularly attractive for higher-dose antidepressants, provided solvent-related tolerability and burst effects are effectively managed [94].

On the other hand, lipid-based depots release drugs primarily through partitioning from an oily or lipid matrix into surrounding aqueous fluids, as well as through lipid reorganization, digestion, or resorption [107]. These mechanisms commonly support depot lifetimes from days to several weeks rather than many months, making them more appropriate for short-to-intermediate dosing intervals. These systems may be particularly suitable for highly lipophilic antidepressants, such as amitriptyline or sertraline, when a 1–4-week dosing interval is clinically sufficient and avoidance of acidic polymer-degradation products is desirable. However, their partition-driven kinetics and comparatively limited duration mean that lipid depots are unlikely to be universal solutions for all antidepressants unless combined with other release-controlling mechanisms in hybrid formulations [105]. Hybrid and emerging systems, including injectable hydrogels and stimulus-responsive depots, combine diffusion through hydrated networks with matrix degradation, erosion, or environmentally triggered changes in network structure [144]. These platforms may offer smoother, more nearly zero-order release profiles and can potentially support co-delivery of multiple therapeutic agents [145]. However, their current limitations relate to formulation complexity, scale-up, sterility assurance, batch reproducibility, long-term safety of crosslinkers or nanomaterials, and limited clinical precedent relative to microspheres, oil depots, and conventional in situ depots [125].

Overall, a cross-platform comparison of release mechanisms suggests that controlling initial burst release is critical for antidepressant LAIs, as excessive peak concentrations may compromise tolerability, and rapid concentration decline may increase the risk of discontinuation symptoms. Platforms that combine diffusion with slow matrix degradation or erosion, particularly microspheres, ISFDs, and biodegradable hydrogels, are the most plausible backbone technologies for achieving week-to-month release [86]. Nanoparticles and lipid systems may instead be more useful for improving solubility, tissue distribution, or intermediate-duration release, or when integrated into hybrid depot designs. Platform selection should ultimately be guided by the antidepressant’s dose requirement, solubility, lipophilicity, stability, desired duration, and intended clinical use [105].

### 4.3. Dose-Loading Capacity and Injection-Volume Constraints

For antidepressant LAIs, achievable drug loading and acceptable injection volume are central determinants of platform feasibility. Many antidepressants require maintenance doses that may be difficult to accommodate in a single monthly or longer duration. PLGA- and PLA-based microspheres can provide moderate-to-high drug loading, but loading depends on drug solubility, polymer composition, manufacturing process, and desired release duration. For partially water-soluble or amphiphilic drugs, drug partitioning into the external aqueous phase during manufacturing can reduce encapsulation, while excessive loading may affect particle morphology, burst release, and injectability. Thus, the injectable dose is limited by both drug loading and the quantity of microspheres that can be delivered through an acceptable needle [62]. Polymeric nanoparticles impose greater dose-capacity constraints as standalone depots because their high surface area can favor faster release, and acceptable injection volumes contain limited total drug mass. They may therefore be more suitable for potent drugs, tissue-distribution modulation, or incorporation as primary vehicles into larger hydrogels or ISFDs [146]. ISFDs on the other hand can accommodate higher absolute doses because a drug may be dissolved or suspended directly in a polymer–solvent formulation, and the dose can be adjusted by varying the concentration or injection volume. Their utility, however, is limited by viscosity, needle injectability, solvent exposure, burst release, and depot integrity [147].

Lipid depots are most suitable for potent, highly lipophilic drugs or lipophilic prodrugs that can dissolve directly in oils or structured lipid vehicles. Their capacity depends on lipid solubility, oil/water partitioning, vehicle viscosity, and tolerable injection volume. Hydrophilic or strongly ionized drugs usually require co-solvents, ion pairing, prodrug design, or more complex lipid architectures [107]. Hybrid hydrogels and composite depots may expand the formulation space by combining a polymeric network with drug crystals, nanoparticles, microparticles, or lipophilic domains. Such architectures can improve retention of poorly soluble drugs, moderate burst release, or enable separate loading of multiple agents; however, they also introduce added complexity in manufacture, characterization, sterilization, and batch-to-batch reproducibility [130].

Overall, dose loading and injection volume establish a practical hierarchy for antidepressant LAI development. ISFDs and optimized microspheres are likely to be the most feasible starting platforms for moderate-to-higher-dose antidepressants, if loading, burst release, and injectability satisfy the acceptance criteria. Lipid-based depots may be particularly suitable for potent and highly lipophilic drugs or prodrugs, whereas nanoparticles are more likely to add value as components of composite systems than as high-capacity standalone depots. Platform-specific strategies including ion pairing, amorphization, polymer selection, solvent optimization, lipidic prodrug design, and hybridization will therefore be necessary to align drug loading with an acceptable injection volume and intended dosing interval.

### 4.4. In Vitro–In Vivo Correlation (IVIVC) and Product Robustness

Establishing an in vitro–in vivo correlation (IVIVC) is a major challenge in LAI development. In vitro release tests are essential for batch release, formulation screening, and stability assessment, but they may not accurately reproduce the physiological conditions experienced by a depot after injection. Local tissue fluid exchange, enzymatic activity, pH, temperature, mechanical stress, inflammation, and depot morphology can all influence in vivo release, particularly for biodegradable polymers, ISFDs, and responsive hydrogels. These factors are particularly important for biodegradable microspheres, ISFDs, and responsive hydrogels, in which drug release may involve an initial burst phase, diffusion through a changing matrix, and later polymer erosion or degradation [148]. Consequently, a formulation that appears to provide stable release in vitro may show a different burst, lag phase, or terminal release profile in vivo [149].

For small-molecule antidepressant LAIs, release testing should be designed to identify clinically meaningful differences in initial burst, lag time, total duration of release, and late-phase drug depletion. Where possible, in vitro methods should be linked to pharmacokinetic data from relevant animal models and subsequently from human studies. Model-informed drug development, including population pharmacokinetic and physiologically based pharmacokinetic modeling, may assist in translating release characteristics into predicted exposure profiles, selecting dose strengths, and evaluating the consequences of delayed or missed doses [138,150].

For antidepressants characterized by short elimination half-life or clinically significant discontinuation symptoms, such as paroxetine and venlafaxine, establishing a practical IVIVC framework is essential [51]. Consequently, the evaluation of a potential LAI must extend beyond its rudimentary assessment of its in vitro release duration. Instead, candidate formulations featuring variations in polymer composition, molecular weight, particle size, drug loading, porosity, or surface-associated drug content must first be analyzed using discriminatory in vitro release methodology [62]. The resulting release profiles should subsequently be correlated with in vivo pharmacokinetic data obtained from designated IM or SC administrations. Crucial pharmacokinetic parameters for this comparative analysis include early peak plasma concentration (Cmax), time to peak concentration (Tmax), area under curve (AUC) during the immediate post-injection phase, maintenance-phase plasma concentrations and kinetics of terminal concentration decline [64]. Hypothetically, a significant initial burst release would increase the likelihood of excessive early systemic exposure, potentially leading to adverse effects. Conversely, accelerated late-phase release or premature depot depletion could result in subtherapeutic drug concentrations prior to the next scheduled administration [139]. For drugs with severe adverse effects, a clinically useful depot should therefore minimize excessive early exposure, avoid a substantial lag phase, maintain adequate exposure across the intended dosing interval and exhibit gradual rather than abrupt terminal decline.

However, directly validated IVIVC examples for small-molecule antidepressant LAIs remain limited as antidepressant-specific depot products are still at an early stage of development [138]. However, preclinical polymeric formulations of antidepressants illustrate the importance of linking formulation characteristics and in vitro release with in vivo drug disposition. For example, chitosan-modified PLGA nanoparticles containing paroxetine have been evaluated for particle size, drug loading, in vitro release, cellular uptake, biodistribution, and pharmacodynamic effects in mice [130]. Venlafaxine-loaded PLGA nanoparticle systems have also been investigated in preclinical studies for controlled release and brain-targeted delivery [151]. However, these findings alone cannot be used for practical understanding of IVIVC, as nanoparticle systems, routes of administration, and experimental objectives may differ from those required for systemic IM or SC LAI depots [77]. Accordingly, a predictive IVIVC must be established separately for each antidepressant platform combination rather than inferred from in vitro release testing alone [66].

Product robustness is equally important because LAIs contain a substantial drug load that may be released over weeks or months. The formulation should maintain consistent performance after storage, transport, resuspension, temperature excursions, and administration through the intended needle and syringe system. It should also be evaluated for sensitivity to variations in injection technique, administration site, patient physiology, and co-administered medications where these could alter absorption or depot integrity [149]. A central concern is dose dumping, unintended rapid release of all or a substantial fraction of the drug load. This may lead to excessive early exposure, toxicity, injection-site complications, and loss of therapeutic coverage later in the dosing interval. Modified-release regulatory guidance identifies dose dumping as an important risk requiring investigation, especially when release may be altered by physiological or formulation-related conditions. For stimulus-responsive systems, robustness testing is particularly critical. Small variations in local pH, ionic strength, temperature, inflammation, or enzymatic activity may unintentionally trigger or suppress release [152]. Although this responsiveness may be therapeutically useful, the system must demonstrate that clinically acceptable biological variation does not cause unpredictable exposure [145]. Therefore, robust, biorelevant release methods and evidence of reproducible in vivo performance will be essential before advanced responsive platforms can be translated into chronic antidepressant therapy.

### 4.5. Manufacturing Feasibility and Scalability

Manufacturing feasibility and scalability differ markedly across LAI platforms and directly influence their suitability for antidepressant development. Polymeric microspheres benefit from decades of industrial experience, with solvent evaporation, phase separation, and spray-drying processes already used for marketed products [153]. Their key quality attributes including particle size distribution, morphology, residual solvents, drug content, and release profile are well defined, and established approaches exist for sterile manufacture, suspension formulation, and container-closure design. This gives microspheres a relatively lower technical risk when adapting the platform to a new antidepressant [154]. Polymeric nanoparticles are more sensitive to small variations in mixing conditions, solvent ratio, surfactant concentration, temperature, and purification steps. These variables can alter particle size, polydispersity, surface properties, drug loading, and release behavior, making scale-up from laboratory nanoprecipitation or emulsification methods challenging [140]. Continuous manufacturing approaches, including flash nanoprecipitation and microfluidics, may improve reproducibility, but nanoparticle products still require extensive control of particle characteristics and batch consistency [155].

ISFDs avoid particle fabrication and can simplify manufacture by using a homogeneous drug–polymer–solvent solution. However, reliable scale-up still requires control of polymer molecular weight, polydispersity, solvent quality, residual impurities, drug solubility, and solution rheology, because minor variation can affect injectability, phase inversion, depot morphology, and release behavior [86]. Lipid-based systems limit a broad range of complexities. Simple oil-based solutions and suspensions can be produced using conventional mixing and aseptic filling processes, with emphasis on drug content, viscosity, stability, and sterility. In contrast, liposomes and lipid nanoparticles require specialized methods such as high-pressure homogenization, microfluidics, extrusion, or controlled solvent-injection processes. Their manufacturing also requires a rigorous assessment of size distribution, surface charge, lipid crystallinity, encapsulation efficiency, physical stability, and, where applicable, lamellarity [156]. Hybrid and stimulus-responsive systems remain the most technically demanding platforms. Their multicomponent architecture, crosslinking reactions, and responsiveness to external or physiological triggers can complicate manufacture, sterilizations, release testing, and batch-to-batch reproducibility. Although these systems offer highly tunable release profiles, they are likely to require longer development timelines and more extensive quality-control strategies before large-scale implementation for antidepressant LAIs [130].

**Figure 8:**
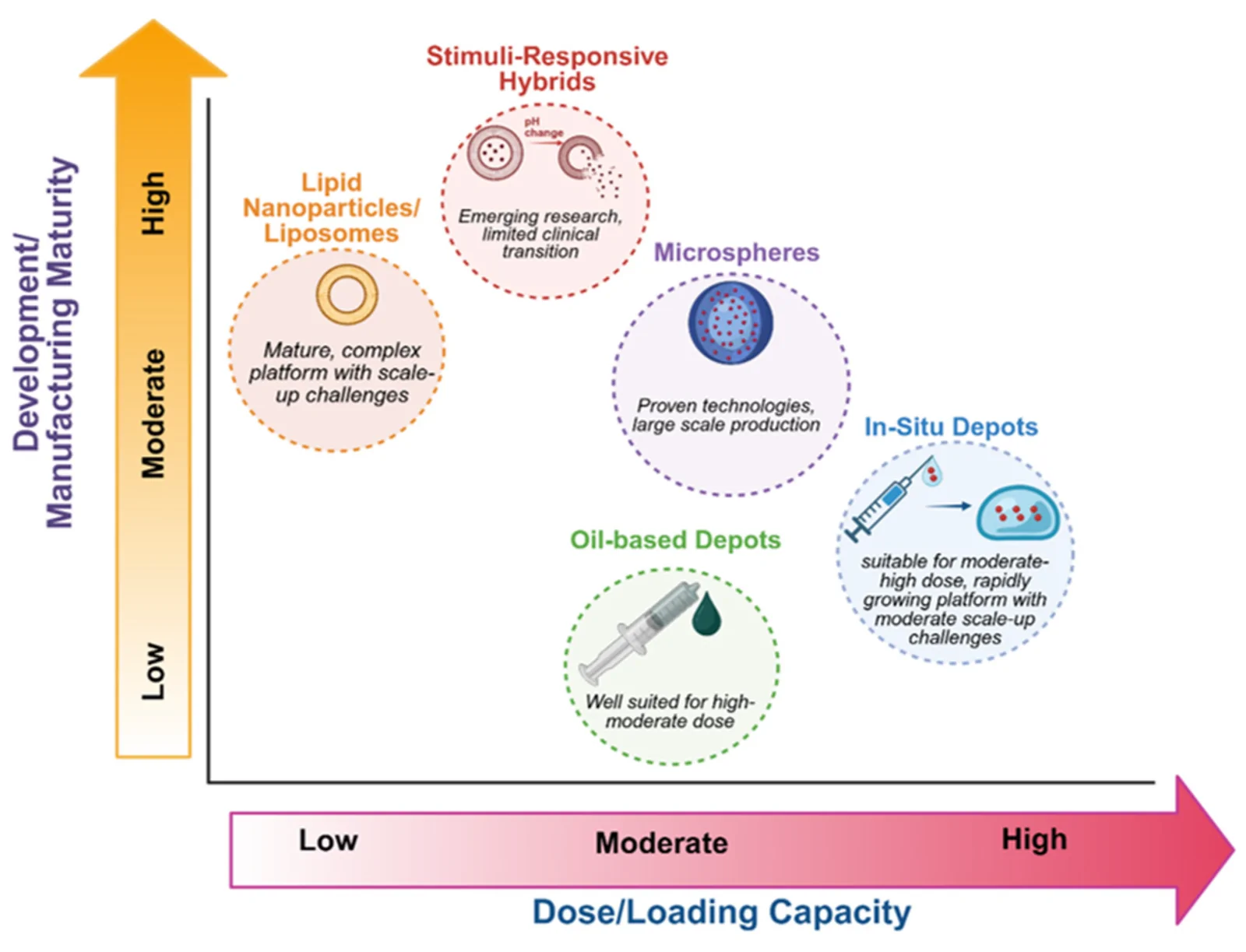
Comparative positioning of LAI platforms by dose-loading capacity and manufacturing maturity [created using Biorender; Garg. S (2026) https://BioRender.com/w4kkrfu].

Overall, microsphere and simple oil-based depots currently offer the most established and scalable manufacturing routes for antidepressant LAIs, whereas ISFDs provide a realistic intermediate option by avoiding particle-processing steps while retaining flexible dose adjustment (Figure 8). Polymeric nanoparticles, liposomes, lipid nanoparticles, and hybrid hydrogels offer greater opportunities for controlled distribution and tailored release but require more demanding process control, characterization, and scale-up strategies. Therefore, platform selection for antidepressant LAIs should balance the desired pharmacokinetic profile against manufacturability and product robustness, with more complex systems justified only when they provide a clear clinical advantage over established microsphere, oil-based, or ISFD technologies.

### 4.6. Route of Administration, Injectability, and Patient Acceptability

The route of administration is a key design consideration for antidepressant LAIs because it affects achievable dose, injection volume, release kinetics, local tolerability, and practical use in routine care. IM administration can generally accommodate larger injection volumes and has substantial precedents for depot medicines, while SC administration may offer easier administration and potentially greater patient convenience. However, SC tissue can be more sensitive to formulation volume, viscosity, and depot-related irritation. Differences in tissue vascularity, injection site, body composition, and injection technique may also contribute to variability in absorption and systemic exposure [157].

Injectability is determined by formulation characteristics such as viscosity, particle size, particle concentration, syringeability, sedimentation, resuspendability, needle compatibility, and the force required for injection. Microsphere suspensions must remain sufficiently uniform during storage and administration to ensure dose accuracy, while highly viscous ISFDs, hydrogels, and lipid formulations may require larger needles or greater injection force [158]. These considerations can limit the polymer concentration, drug load, and injection volume that can be used, even where a formulation is otherwise pharmacokinetically viable.

Patient acceptability is particularly important for antidepressant LAIs because treatment may be used for extended periods in individuals who are well enough to make preference-sensitive decisions about their care [150]. Pain, injection-site nodules, visible swelling, prolonged soreness, frequent clinic attendance, perceived stigma, and lack of flexibility in stopping treatment may all reduce uptake and persistence. Therefore, the preferred platform should not simply achieve the longest possible dosing interval. It should provide a clinically useful interval while maintaining acceptable injection volume, tolerability, and ease of administration [159]. For first-generation antidepressant LAIs, a desirable target may be a formulation compatible with standard SC or IM administration, a manageable injection volume, and a dosing interval that balances adherence benefits against the risk of prolonged adverse effects or limited reversibility. Patient-reported outcomes, injection-site tolerability, administration time, and preference relative to oral therapy should be incorporated early into clinical development rather than evaluated only after pharmacokinetic feasibility has been established [160].

### 4.7. Safety and Regulatory Considerations

Safety assessment for LAI platforms should focus on excipient biocompatibility, local tissue tolerability, and systemic toxicity. For antidepressant LAIs, these issues are especially important because treatment may continue for years in patients who are often clinically stable between episodes. Therefore, even non-serious injection-site reactions may adversely affect adherence and acceptability.

PLGA/PLA microspheres and several conventional lipid excipients have an established history of parenteral use, which reduces uncertainty relative to entirely novel materials [161]. Nevertheless, polymer properties, depot size, degradation rate, residual solvents, and injected dose can influence local tolerability. PLGA degrades to lactic and glycolic acid, but local accumulation of these acidic degradation products can lower pH and contribute to inflammation or foreign-body reactions if the formulation is not appropriately designed [162]. Nanoparticle-based systems introduce additional regulatory complexity. Although their individual polymer or lipid components may be familiar, the assembled nanostructure can alter biodistribution, cellular uptake, clearance, immune interactions, and off-target tissue exposure. Consequently, long-term repeat-dose development would need robust evidence on biodistribution, persistence, degradation, immunogenicity, and potential accumulation in non-target organs, rather than relying solely on the prior safety record of the constituent excipients [140].

For ISFDs, the principal safety concern shifts to the organic solvent and the phase-inversion process after injection. The development process should establish that systemic solvent exposure is acceptable and that solvent exchange, depot formation, and repeated administration do not cause unacceptable pain, necrosis, persistent inflammation, fibrosis, or other injection-site complications. The search for less-toxic solvent systems reflects the importance of this issue for PLGA-based ISFDs [163]. Oil-based depots and simpler lipid systems may benefit from established parenteral excipients and comparatively straightforward safety packages. However, high local drug or oil concentrations, large injection volumes, depot viscosity, and slow absorption can still cause pain, nodules, or variable exposure. Liposomes, lipid nanoparticles, hybrid depots, and stimulus-responsive systems require broader characterization because their particle structure, surface properties, degradation products, and novel materials may create safety considerations not predicted by the individual components alone [156].

Regulatory development should therefore combine systemic toxicology with formulation-specific local-tolerance studies, preferably through the intended clinical route and using a formulation representative of the final product. Regulatory guidance for parenteral products emphasizes evaluating local tolerance as part of general toxicity studies and assessing unintended injection-site exposure wherever relevant [164]. In addition to the formulation safety, LAI antidepressants may raise safety considerations related to patient autonomy and limited reversibility, demanding a predefined safety-management pathway. Unlike oral medicines, depot formulations continue releasing drugs for weeks or months after injection, limiting the ability to immediately stop the treatment if adverse effects, clinically significant drug interactions or changes in patient preference occur. This makes informed consent, confirmation of prior oral tolerability, and clear discussion of pros and cons of prolonged drug exposure particularly important; careful precautions must be taken for formulations such as dispersed microspheres or non-retrievable systems where depot removal is not feasible post administration [165].

Limited reversibility of the LAI depots also restricts the flexibility required when antidepressant treatment needs dose adjustment, switching because of inadequate response or adverse effects, or planned discontinuation. Clinical guidance recommends individualized, stepwise dose reduction when stopping antidepressants, with monitoring for withdrawal symptoms and recurrence of depression [166]. Stopping further LAI injections does not immediately stop exposure from the administered depot, while initiating a replacement antidepressant during residual depot release may complicate interaction management [167]. For example, fluoxetine is a drug with a long elimination half-life and requires prolonged exposure for up to several weeks for achieving the desired plasma concentration of its therapeutic profile. Likewise, post-discontinuation of fluoxetine, a five-week interval as a washout period is suggested prior to initiation of any other antidepressants [168]. This pharmacokinetic behavior suggests that for future fluoxetine LAIs, the additional depot-release period would require separate evaluation rather than assuming that the oral washout interval remains sufficient. Moreover, a declining depot release for any drug should not be assumed to provide an immediate decrease in plasma concentrations [157]. These considerations support evaluating LAI antidepressants initially in patients with an established, effective, and tolerated maintenance regimen rather than those requiring frequent treatment changes.

Also, as the LAI systems act as a continuing source of drug input, CYP-mediated interactions require particular attention. CYP inhibitors and inducers generally alter the metabolism and systemic clearance of drugs released from the depot, rather than directly changing [169,170] the physical release rate from the depot reservoir [171]. Thus, a CYP inhibitor may reduce clearance and cause sustained elevation of systemic drug exposure while the depot continues to release the drug, whereas a CYP inducer may increase clearance, reduce exposure, and contribute to treatment failure, symptom recurrence, or discontinuation-like symptoms [169]. Clinical development should therefore characterize the CYP and transporter pathways of each candidate antidepressant, assess relevant inhibitors and inducers through in vitro studies and clinical interaction studies where required, and use population pharmacokinetic or physiologically based pharmacokinetic modeling where appropriate [172]. When a clinically important inhibitor or inducer is introduced after injection, management should follow product-specific guidance and may include replacement of the interacting drug, enhanced clinical monitoring, therapeutic drug monitoring where validated, and adjustment of subsequent—not already administered—LAI doses or intervals [169,170].

Evidence from LAI antipsychotic practice also suggests that injectable treatment may be perceived as restrictive if patient choice and shared-decision-making are not adequately protected. Future LAIs should therefore be introduced through a patient-centered process that prioritizes voluntary informed choice, patient preference, and a clear plan for adverse effects, missed doses, interactions, and discontinuation [173].

Overall, established microsphere and simple lipid-depot platforms are likely to present the clearest regulatory path for antidepressant LAIs because their excipients and manufacturing approaches have substantial precedents. More complex nanoparticle, hybrid, and responsive systems may still be valuable where they offer a meaningful pharmacokinetic or tolerability advantage, but their development must demonstrate long-term local and systemic safety, acceptable reversibility, and patient-centered clinical implementation for chronic use in MDD populations.

## 5. Translational Barriers and Future Opportunities

Translating LAI antidepressants into clinical practice will require overcoming interconnected dose, formulation, safety, administration, regulatory, and patient-selection challenges. These barriers differ from those of established LAI antipsychotics because many antidepressants have higher daily dose requirements and narrower acceptable limits on injection volume, and are intended for long-term use in a preference-sensitive population. LAIs are complex drug products: relatively small changes in material attributes, manufacturing conditions, or device performance can alter release and systemic exposure [174].

### 5.1. Dose Requirements

Most marketed LAI antipsychotics deliver relatively potent drugs at doses that remain manageable within depot size and injection volume when formulated as microspheres, in situ depots, or crystalline suspensions. For example, commercially available paliperidone palmitate monthly formulations (150–234 mg in 1–2 mL) or aripiprazole (441–882 mg in 1.5–2.5 mL) [175] stay within standard IM tolerability thresholds, even for 3- to 6-month intervals. In contrast, many antidepressants require higher daily maintenance doses (e.g., sertraline 100–200 mg/day [176], fluoxetine 40-80 mg/day [168]), implying 3 to 10 gms total content for equivalent monthly coverage, substantially exceeding current depot capacities. This amplifies constraints on drug loading, with high injection volumes > 3 mL, and increases local tolerability challenges. Therefore, early antidepressant-LAI candidates are likely to favor molecules with relatively low dose requirements, adequate lipophilicity, suitable stability, and a therapeutic window compatible with sustained exposure. Higher-dose agents may require shorter dosing intervals, multiple injection sites, highly concentrated depots, prodrug strategies, or future high-capacity delivery technologies. Drug solubility, partition coefficient, particle size, and half-life are all important determinants of LAI feasibility [177].

### 5.2. Safety of Solvents, Excipients, and Depot Materials

The choice of excipient is the central barrier across various LAI delivery platforms. PLGA/PLA-based microspheres have extensive parenteral route approval but generate acidic degradation products (including oligomers, lactic/glycolic acids) that can trigger local inflammation, impair drug stability, and cause pain at the injection site [178,179]. Similarly, ISFDs rely on solvents like N-methyl-2-pyrrolidone (NMP), which must stay within ICH Q3C(R9) PDE limits (53 mg/day) to avoid tissue irritation or systemic toxicity during phase inversion [180]. Lipid/oil vehicles use established excipients, but high drug/oil concentrations can still produce pain, nodules, and variable absorption [181]. Nanoparticles raise concerns about long-term tissue accumulation, immunogenicity, and off-target deposition, particularly for chronic dosing [146]. These challenges make selection of excipients a critical choice while designing LAI for antidepressants. As the antidepressants are used for long periods in relatively healthy populations, excipient tolerability thresholds must be given primary importance with respect to their acceptable limits, and this narrows down the formulation space for designing [182].

### 5.3. Injection-Volume Constraints and Patient Acceptability

Clinical data confirms that IM injections exceeding 2 to 3 mL per site are associated with increased pain intensity and reduced patient acceptability, thereby driving depot formulations toward maximizing drug content per unit volume [183]. The higher total dose requirements of antidepressants translate these volume constraints into harsh drug-loading targets exceeding 15 to 20% *w*/*w*, favoring high-capacity in situ-forming depots and concentrated microsphere suspensions over lower-capacity nanoparticles [184]. SC administration imposes even tighter volume limitations of approximately 1 mL per site and demonstrates greater sensitivity to formulation-related pain and depot consistency [185]. High dose requirements may necessitate higher drug loading, increased formulation concentration, shorter dosing intervals, or multiple injection sites, although increasing concentration may compromise injectability or release performance [109].

For example, if sertraline has an oral starting adult dose of 50 mg/day, with 50% oral bioavailability with a 30-day dosing interval, a nominal LAI depot would require 1500 mg of total dose to be loaded [186]. For a hypothetical microsphere suspension containing 500 mg of dry microspheres/mL, drug loading of 10–30% *w*/*w* would provide active-drug concentrations of 50–150 mg/mL, requiring an injection volume of up to 10 mL. At 20% loading, reducing the dosing interval to 7 days would reduce the nominal drug requirement to 350 mg and volume to 3.5 mL. However, these calculations are based on simple dose-exposure (Equation (1)) [187] and mass-concentration (Equation (2)) [109] equations mentioned below, but the actual dose conversion must account for differences in oral and depot bioavailability, clearance, release completeness, and the desired concentration–time profile.

Bioavailability-adjusted dose estimation:


(1) $$M_{\mathrm{depot}}=\;D_{\mathrm{oral}}\;\times \;T\;\times \;\frac{F_{\mathrm{oral}}}{F_{\mathrm{LAI}}}$$

Here,

*D_oral_* is oral dose (mg/day);

*T* is the dosing interval (days);

*F* is systemic bioavailability.

Injection-volume estimation based on mass-concentration:


(2) $$V_{\mathrm{inj}}=\;\frac{M_{\mathrm{depot}}}{C_{\mathrm{drug}}}=\;\frac{M_{\mathrm{depot}}}{C_{\mathrm{LAI}}\;\times \;L}$$

Here,

*V_inj_* is the volume to be injected;

*M_depot_* is the mass of the formulation (mg);

*C_drug_* is the concentration of drug required (mg);

*C_LAI_* is the concentration of dry microspheres in the final suspension (mg/mL);

*L* is the drug-loading fraction of microspheres.

Moreover, the achievable loading and suspension concentration must be determined alongside the viscosity, syringeability and release reproducibility, and local tolerability of the formulation. Consequently, a low daily dose alone does not establish feasibility, and dose, dosing interval, loading and formulation concentrations must be considered together during development [109]. Patient acceptability should also be assessed alongside formulation feasibility. Injection pain, treatment frequency, clinic attendance, and limited reversibility may influence the willingness to initiate and continue treatment. Patients with depression may also have less familiarity with ongoing injectable therapy than individuals treated for psychotic disorders. Therefore, patient preference and perceived treatment benefit should be incorporated into development and shared decision-making [188].

### 5.4. Regulatory Expectations for LAIs and Complex Depots

LAIs, microspheres, ISFDs, liposomes, nanoparticles, and implantable products are generally treated as complex drug products because their performance depends on interrelated formulation, manufacturing, administration, and biological variables. Delivery system development must define and control critical quality attributes such as drug loading, particle-size distribution, morphology, polymer molecular weight, residual solvents, viscosity, release profile, sterility, stability, and syringe-needle compatibility [187]. PLGA-based microspheres and ISFDs require robust IVIVC models, detailed characterization of release mechanisms and strict control of critical material attributes. In vitro release methods must be sensitive enough to detect meaningful manufacturing changes and predictive enough to support bioequivalence, an essential requirement for any future generic products [188]. For antidepressant LAIs, clinical development should demonstrate not merely prolonged exposure but a meaningful patient benefit relative to oral treatment. Potential benefits might include improved adherence, reduced pharmacokinetic fluctuation, lower relapse risk, or improved patient-reported outcomes in a clearly defined population. A prolonged depot should not be assumed to be safer than oral therapy, because it may also prolong adverse effects or reduce the ability to rapidly adjust treatment [174].

### 5.5. Opportunities for Combination Therapy and Regimen Simplification

LAI antidepressants could potentially support regimen simplification for patients requiring long-term adjunctive treatment. In depression with psychotic features, bipolar depression, treatment-resistant depression, or significant comorbid anxiety, coordinated administration of an antidepressant LAI with another long-acting treatment could reduce daily pill burden and improve certainty of exposure. Long-acting delivery systems can reduce dosing frequency and may improve adherence, while long-acting microsphere systems have been proposed as platforms for multi-drug fixed-dose combinations [189].

However, combining two active agents within a single depot is substantially more difficult than administering separate LAIs. The drugs must have compatible drug loading, solubility, stability, dose requirements, release kinetics, and intended dosing intervals. In mixed-payload microspheres, initial burst release, phase separation, and different release mechanisms may make it difficult to maintain a consistent drug ratio [108]. Similarly, co-precipitation within ISFDs can lead to non-uniform drug distribution and preferential release of one component. A fixed-dose combination also reduces the ability to adjust, stop, or switch one component independently when adverse effects, drug interactions, changing symptoms, or diagnostic uncertainty arise. Regulatory standards require evidence that each component contributes to the claimed therapeutic effect and that the fixed dose, dosing frequency, and duration are safe and effective for the intended population [190].

For this reason, coordinated schedules involving separate long-acting products may be more plausible in the near term than co-encapsulated antidepressant-adjunctive depots. A single-injection combination should be pursued only where there is a stable and clinically justified dose ratio, compatible pharmacokinetics and release profiles, an acceptable shared safety profile, and a clear advantage over separate administration.

### 5.6. Personalized Medicine, Patient Selection, and Emerging Materials

The initial clinical role of LAI antidepressants is likely to be relevant for carefully selected patients rather than all individuals with depression. Potential candidates may include patients with recurrent MDD, repeated early discontinuation of oral therapy, difficulty sustaining daily medication routines, or a documented stable response and tolerability to the oral form of a proposed LAI drug. This approach is informed by experience with LAI antipsychotics, for which non-adherence and recurrent relapse are important reasons to consider sustained-delivery treatment; however, the suitability of this model for antidepressants will need direct clinical evaluation rather than simple extrapolation [191] These proposed populations are conceptual targets for future development and are not supported by direct antidepressant-LAI clinical trial evidence.

The potential suitability of LAI antidepressants is also likely to differ across different depression conditions. Recurrent MDD requiring maintenance treatment may represent the most plausible initial setting, particularly for patients with established oral response and tolerability who experience persistent non-adherence or early discontinuation. In contrast, treatment-resistant depression and depression with psychotic features may require more cautious evaluation as treatment regimens often involve augmentation, polypharmacy, and a need for flexible dose adjustment [192]. Bipolar depression, perinatal depression, seasonal affective disorder, and first-episode or clinically unstable depression should also be approached cautiously because of mood-switch risk, pregnancy or lactation considerations, time-limited symptom patterns, diagnostic uncertainty, and the limited reversibility of depot exposure [193]. Direct clinical evidence for antidepressant-specific LAIs in these populations remains limited; therefore, these distinctions should guide future research and patient selection rather than be interpreted as clinical recommendations.

Conversely, an LAI antidepressant may be less appropriate for patients who require rapid dose adjustment, have not yet demonstrated oral tolerability, experience unpredictable adverse effects, have substantial pharmacokinetic drug-interaction risk, or strongly prefer treatment that can be stopped immediately. Patient selection should therefore consider prior response, dose requirement, relapse pattern, hepatic and renal function, co-medication, access to injection services, needle preference, and informed choice. The prolonged duration of depot exposure means that shared decision-making and a successful oral lead-in period would be particularly important before initiating treatment [186]. Before the first LAI injection, patients should demonstrate tolerability to the same oral active drug, or a scientifically justified oral equivalent, at a stable exposure relevant to the proposed LAI regimen. This pre-requisite should confirm the absence of any clinically significant adverse effects, a stable clinical condition, and an acceptable drug-interaction profile. Pre-injection assessment should also include a review of prescription medicines, over-the-counter products, herbal medicines, alcohol and substance use, prior adverse drug reactions, hepatic and renal function where relevant, and the presence of CYP inhibitors or inducers [161, 188] Baseline symptom severity, suicide risk, and patient understanding of prolonged exposure, limited reversibility, potential injection-site reactions, missed-dose procedures, and the adverse-event pathway should be documented before treatment. Patients requiring rapid dose adjustment, those with unresolved or unpredictable adverse reactions to oral therapy, and those receiving unavoidable strong CYP inhibitors or inducers may be unsuitable candidates for an initial LAI formulation [169].

Digital tools may eventually assist patient selection and monitoring, but they should be viewed as supportive rather than definitive. Smartphone and wearable data can continuously capture behavioral indicators relevant to depression, including sleep, mobility, physical activity, communication patterns, and device use; systematic-review evidence suggests that these measures may help track symptom changes or predict relapse and exacerbation. However, variable engagement, privacy considerations, incomplete data, and uncertain individual-level predictive performance remain important limitations [194].

Emerging delivery materials may expand the range of antidepressants that can be formulated as LAIs. Stimulus-responsive hydrogels, hybrid polymer–lipid depots, nanocomposites, and more biocompatible polymer chemistries may improve control of burst release, depot integrity, drug loading, and local tolerability. Stimulus-responsive hydrogels can alter swelling, network permeability, or drug release in response to factors such as pH, temperature, ionic strength, enzymes, or inflammatory signals [195]. Nevertheless, these technologies remain at an early translational stage as their in vivo degradation and release may be difficult to predict because material properties interact with dynamic physiological conditions, while potential immune responses, crosslinker toxicity, reproducible manufacture, sterilization, and scale-up must be addressed before clinical use [196] Accordingly, practical initial antidepressant LAIs are likely to use established materials and platform technologies, with patient selection based on demonstrated oral response, adherence-related need, safety, and preference. Advanced responsive and hybrid materials may provide important longer-term opportunities, but their use will depend on showing predictable release, long-term biocompatibility, manufacturing reproducibility, and a meaningful advantage over conventional depot systems.

Overall, the translation of LAI antidepressants will depend on more than achieving sustained drug release. A successful product must accommodate a clinically relevant dose within an acceptable injection volume, demonstrate predictable pharmacokinetics and local tolerability, have scale-up feasibility and provide a meaningful advantage over oral treatment for an appropriately selected patient group (as discussed in Table 5). Established microsphere, lipid, and ISFD technologies are likely to offer a practical initial pathway, whereas hybrid and stimulus-responsive systems have scope as future long-term options that require further evidence of safety, reproducibility, and clinical value.

## 6. Artificial Intelligence and Machine Learning in LAI Formulation Development

The development of LAI formulations is inherently resource-intensive: conventional workflows rely on iterative “formulate-and-test” cycles in which polymer chemistry, drug loading, particle size, and release kinetics are tuned empirically, often over several years of preclinical effort [197]. Machine learning (ML) and broader artificial intelligence (AI) methods are emerging as tools to accelerate this process by learning the complex, non-linear relationships between formulation variables and drug-release kinetics directly from experimental data [198]. For LAI antidepressants where the candidate molecule must be matched to an appropriate depot platform against tight constraints on dose, injection volume, and release duration, such predictive models could shorten development time, minimize in vitro and in vivo testing, and guide platform selection before committing to preclinical studies [199].

Bannigan et al. demonstrated a data-driven approach to polymeric LAI design, training and benchmarking eleven ML algorithms, including multiple linear regression, partial least squares, decision tree, random forest, light gradient boosting machine (LGBM), extreme gradient boosting, support vector regression, k-nearest neighbors, and neural networks, to predict fractional drug release from polymeric LAIs [200]. The LGBM model demonstrated the highest predictive accuracy and successfully guided the design of novel formulations. The authors emphasize that while data-driven approaches meaningfully reduce development time and cost, their predictive capacity remains constrained by the quantity, quality, and standardization of available release data [200].

A more integrated computational strategy is offered by Formulation LAI, a closed-loop framework combining ML, physiologically based pharmacokinetic (PBPK) modeling, and molecular dynamics (MD) simulations [197]. Using the commercial PLGA-based injectable Zilretta^®^ as a benchmark, PBPK/pharmacodynamic modeling defined target in vitro release profiles to maximize therapeutic exposure; ML guided formulation optimization, and MD simulations resolved the drug–polymer interactions. In rat pharmacokinetic studies, the optimized ISFD achieved a 34% increase in drug exposure relative to the commercial suspension. The framework was projected to compress preclinical development from a traditional 5–7 years to 1–2 years, shifting the paradigm from “formulate-and-test” to “compute-and-validate,” an approach directly suited to the tight constraints of antidepressant LAIs.

Complementing these predictive efforts, explainable ML was applied to a dataset of LAI formulations to model both early (24, 48, and 72 h) and complete release profiles. The model achieved strong correlation (r > 0.65) at 72 h and an F1-score of 0.87 for release-profile classification [199]. Notably, its time-independent ML framework outperformed conventional time-dependent approaches in predicting delayed biphasic and triphasic curves. Shapley additive explanations identified the primary material characteristics that most strongly govern early and complete release kinetics and filled gaps in prior in vitro and ML-based studies. For LAI antidepressants, where burst or incomplete release compromises safety and sustained efficacy, these explainable models help define acceptable release design spaces early in development.

Together, these studies demonstrate that ML/AI has transitioned from an exploratory concept into a practical driver of LAI formulation, supporting candidate screening, release kinetics forecasting, in vitro–in vivo correlation (IVIVC), and rational platform design. For antidepressant LAIs, predictive modeling offers a clear path to reconcile high dose demands with volume and duration limits while de-risking protracted development cycles. Realizing this potential, however, requires addressing key bottlenecks: dataset scarcity and fragmentation, limited generalizability across polymer classes, and the ongoing need for rigorous external and in vivo validation. Standardized release-testing protocols, open data sharing, and deep integration with PBPK modeling will be essential to translate these computational tools into clinically viable LAI antidepressants.

## 7. Conclusions

Long-acting injectable antidepressants represent a favorable but still largely unexplored approach to improving the delivery of antidepressant therapy. By reducing dependence on daily oral dosing, LAIs could potentially support adherence, reduce pharmacokinetic fluctuations, and provide more consistent therapeutic exposure for patients with recurrent depression, repeated treatment discontinuation, or difficulty maintaining regular oral medication. However, the feasibility of this approach will vary substantially between antidepressant molecules and cannot be assumed solely from the success of LAI antipsychotics.

Candidate selection for early LAI antidepressant development should not be based on maintenance dose alone. The most suitable candidates are likely to combine a feasible total drug mass and injection volume with adequate polymer or lipid compatibility, controllable loading and release, chemical stability during manufacture and depot residence, an acceptable therapeutic window, manageable CYP-mediated interaction risk, predictable pharmacokinetics, and demonstrated oral efficacy and tolerability. The proposed decision framework provides a feasible starting point for matching antidepressant physicochemical and pharmacological characteristics to the most appropriate depot platform, while recognizing that each candidate–platform combination requires experimental validation. Successful development will require careful matching of a drug’s dose requirement, lipophilicity, solubility, ionization behavior, stability, metabolic interactions, and therapeutic window to an appropriate delivery platform.

Polymeric microspheres and conventional ISFDs are reasonable initial platforms for investigation as they are widely accepted marketed products with sustained release, parenteral use, manufacturing, and regulatory development approved. Their prioritization reflects this platform’s maturity rather than demonstrated superiority for antidepressants. Microspheres permit formulation-dependent adjustment of release through polymer and particle properties, but may be constrained by encapsulation efficiency, burst release, suspension injectability, and manufacturing complexity. ISFDs, on the other hand, avoid preformed-particle manufacture and can accommodate dissolved or suspended drugs, although loading and injection volume remain limited by drug solubility, viscosity, solvent exposure, burst release, and local tolerability. Lipid/oil systems are equally plausible alternatives for compatible lipophilic drugs or prodrugs, particularly where adequate concentration and reproducible absorption can be achieved. Hydrogels and emerging hybrid systems offer additional release-design flexibility but generally require greater validation of safety, reproducibility, sterilization, scale-up, and in vivo performance compared to other platforms. Accordingly, platform selection should balance achievable loading and injection volume, release control, manufacturability, tolerability, and clinical precedent rather than favoring one technology universally.

Ultimately, the future of LAI antidepressants lies not in directly replicating existing antipsychotic depot formulations, but in developing patient-centered products that offer a clear advantage over administration. Initial success is most likely to arise from established depot technologies applied to carefully selected, lower-dose and formulation-compatible antidepressant candidates. Emerging hybrid and responsive materials may expand these possibilities over time, provided they can demonstrate reproducible manufacture, long-term biocompatibility, controlled release, and meaningful clinical benefit.

## Figures and Tables

**Table 1:** Types of depression.

S. No	Type of Depression	Symptoms	Ref.
1.	Major depressive disorder (MDD)	Intense low mood	[7]
2.	Melancholic MDD	Affecting physical and emotional behavior	
	Psychological MDD	Delusions, hallucinations, or paranoia	
3.	Bipolar disorder	Extreme mood changes or occurrence episodes of mania or hypomania alternating with depression	[8]
4.	Cyclothymic disorder	Emotional reactivity and affective dysregulation Mostly due to genetic inheritance	[9]
5.	Dysthymic disorder	Smoldering mood disturbance for at least 2 years in adults and transient periods of normal mood	[10]
6.	Seasonal affective disorder	Seasonal pattern of mood changes or persistent sad often lasting about 4–5 months out of a year	[11]
7.	Perinatal depression	Depression during pregnancy around childbirth or within the first year post-partum	[12]

**Table 2:** Commercially available LAI formulations for antipsychotics.

Product Name	Drug	Polymer Used	Delivery System	Route of Administration	Duration of Drug Release	Ref.
Perseris^®^	Risperidone	Poly (DL-lactide-co-glycolide)	Implantable system	SC	1 Month	[29,30]
Zyprexa Relprevv^®^	Olanzapine pamoate	Carboxymethylcellulose	Nanocrystal suspension	IM	2–4 weeks	[31,32]
Invega Sustenna^®^	Paliperidone palmitate	Polysorbate 20, PEG 4000	Nanocrystal suspension	IM	1 Month	[33]
Fluanxol Depot^®^	Flupentixol decanoate	Fractionated Coconut Oil	Oil-based formulation	IM	2–4 weeks	[34]
Vivtrol^®^	Naltrexone	Polylactide-co-glycolide (PLG)	Micro-encapsulation	IM	4 weeks	[35]
Risperdal Consta^®^	Risperidone	Polyglactin	Micro-encapsulation	IM	2 weeks	[36]

**Table 4:** Marketed lipid-based LAI formulations.

Product	API	Vehicle	Indication	Dosing Interval	Key Notes	Ref.
Haldol Decanoate^®^	Haloperidol	Sesame oil	Schizophrenia, chronic psychosis	4 weeks (typical)	Classic oil-based LAI Reduces relapse vs. oral Model for CNS oil depots	[112]
Fluphenazine decanoate	Fluphenazine	Sesame oil	Schizophrenia	2–4 weeks	Early LAI antipsychotic Simple oil solution with long clinical history	[26]
Clopixo^l®^	Zuclopenthixol	Cotton seed oil	Schizophrenia, psychosis	1–4 weeks	Widely used depot Demonstrates flexible interval dosing from oil-based system	[27]
Flupentixol decanoate	Flupentixol	Vegetable oil	Schizophrenia, other psychosis	2–4 weeks	Supports stable symptom control and adherence	[28]
Depo- Testosterone^®^	Testosterone	Cotton seed oil	Hypogonadism	2–4 weeks	Illustrates oil depot platform for endocrine indications	[37]
Faslodex^®^	Fulvestrant	Castor oil	Hormone- positive breast cancer	Monthly	High-dose oil depot Important precedent for highly lipophilic drugs	[113]

**Table 5:** Summary of key translational barriers to LAI antidepressant development and potential mitigation strategies.

Translational Barriers	Critical Antidepressant Category	Relevance to Specific Antidepressant Category	Potential Mitigation Strategies	Suitable/Challenging Delivery Platforms
High dose requirements	SSRIs, SNRIs with moderate–high doses (such as sertraline, fluoxetine, duloxetine, venlafaxine)	A monthly or longer duration LAI would require a large total drug mass which may exceed the practical injection-volume and depot capacity limits	Select lower-dose or highly potent candidates Use shorter dosing intervals Optimize loading concentration Consider pro-drug or ion-pairing approach Assess whether multiple injections are acceptable	Higher capacity ISFDs and concentrated microsphere suspensions may be explored Standalone nanoparticles are generally less practical for high-dose drugs
Hydrophilic, ionization, or low loading efficiency	Hydrophilic or amphiphilic drugs (such as venlafaxine and paroxetine)	Drug partitioning into aqueous phase during manufacture, especially for microspheres, can reduce encapsulation High aqueous solubility may also increase burst release	Optimize pH, ion pairing, drug–polymer affinity, amorphization, and process conditions Compare feasibility of LAI technology in early stages	PLGA/PLA-based microspheres and polymeric nanoparticles are particularly affected ISFDs may provide greater flexibility when the drug is soluble and stable in polymer–solvent system
Lipophilic drugs	Lipophilic TCAs (amitriptyline, nortriptyline, imipramine, clomipramine; potentially mirtazapine, trazodone, or sertraline)	Lipophilic drugs may dissolve or remain suspended at useful concentrations in oils or structured lipid vehicles; however, depot absorption can be variable	Screen oil solubility, viscosity, partition behavior, depot persistence, injection volume, and local tolerance; consider prodrug design where appropriate Confirm reproducible absorption in vivo	Oil-based and structured lipid depots, including lipid-based ISFD systems Polymeric microspheres remain possible where polymer compatibility and loading are satisfactory
Initial burst and end-of-dose decline	Short-half-life or discontinuation-prone antidepressants (such as venlafaxine, paroxetine, desvenlafaxine)	Excessive early release may increase adverse effects Late-phase release may decline reducing efficacy Contributes to discontinuation symptoms	Minimize surface associated drug Optimize particle morphology and polymer composition and control phase inversion IVIVC/PK studies to evaluate early Cmax, trough concentrations and terminal decline	PLGA microspheres, ISFDs, and hydrogels can be of choice with careful control of surface drug, porosity, polymer degradation and solvent phase inversion
CYP-mediated interaction risk	CYP2D6-sensitive or CYP-inhibiting drugs (paroxetine, fluoxetine, venlafaxine, duloxetine, sertraline, citalopram)	CYP inhibitors can increase systemic exposure by reducing clearance, while inducers can decrease exposure and risk symptom breakthroughs The effect may persist while the depot continues releasing drug	Map CYP and transporter pathways Screen prescription, OTC, and herbal products before every injection, avoid strong inhibitors/inducers when feasible Use clinical DDI studies and population PK/PBPK modeling Define product-specific management for post-injection interactions and future-dose adjustment	All systemic LAI platforms, as it is a critical aspect of pharmacokinetics after release rather than a physical depot-release mechanism
Limited reversibility	All antidepressant candidates with high concerns for drugs with difficult-to-manage adverse effects, significant DDI liability, or discontinuation vulnerability (venlafaxine, paroxetine	Drug release may continue for weeks or months after treatment discontinuation, making adverse effects or interactions difficult to manage	Confirm oral efficacy and tolerability before injection Select drugs with wide therapeutic windows Develop missed-dose and adverse-event management plans	Most relevant to microspheres, ISFDs, lipid/oil depots, hydrogels, and other non-retrievable systems
Local tissue tolerability	High-dose candidates (such as sertraline, fluoxetine, duloxetine, and venlafaxine)	Repeated injections may cause pain, nodules, inflammation, fibrosis, or reduced acceptance	Minimize volume and viscosity; select the route based on feasible dose volume Optimize needle/syringe compatibility and injection force Perform repeat-dose local-tolerance and patient-acceptability studies	High-load microsphere suspensions, ISFDs, hydrogels, and oil depots SC systems are generally more volume-constrained than IM systems
Solvent and excipient safety	For all candidates	Organic solvents using ISFD and novel excipients may cause local or systemic toxicity	Selected lower-toxicity solvents Establish residual solvent control Use excipients with parenteral grade and approval Perform biodistribution and degradation studies	Critically for ISFDs and lipid-based depots as major vehicles comprising solvent
Manufacturing and scale-up	All candidates	Small changes in materials or processing may alter particle properties, drug loading, depot formation, or release behavior	Apply quality-by-design principles Tightly control critical material attributes Develop scalable manufacturing methods and discriminating release assays	Microspheres, nanoparticles, liposomes, hybrid systems, and ISFDs Technical complexity increase from simple oil depots to responsive systems
Predictive release testing and IVIVC	Candidates showing early- and late-exposure-dependent effects (including venlafaxine and paroxetine)	In vitro release may not accurately reproduce depot behavior in vivo, limiting prediction of human pharmacokinetics	Develop discriminatory and biorelevant release method Relate in vitro profiles to animal and human PK Evaluate *C*_max_, *T*_max_, partial AUC, trough concentration, and end-of-interval exposure Apply population PK/PBPK modeling	Microspheres, ISFDs, hydrogels, and hybrid systems
Regulatory complexity	All candidates	LAIs are complex products requiring extensive quality, safety, pharmacokinetic, and product-performance evidence	Engage regulators early Define critical quality attributes Establish robust analytical characterization Justify clinical benefit relative to oral treatment	Relevant to all LAI platforms
Patient acceptability	All candidates	Injection pain, needle anxiety, stigma, clinic visits, and treatment inflexibility may limit uptake and persistence LAIs may not suit patients needing rapid titration, frequent dose changes, or immediate reversibility	Use patient-centered product design; minimize volume and injection frequency Assess patient-reported outcomes Support shared decision-making and reliable clinic access Target patients with demonstrated oral response, recurrent illness, adherence-related need, and informed preference for sustained treatment	Relevant to all LAI platforms High-volume, painful, high-viscosity, or non-retrievable depots may reduce acceptability
Combination therapy	For treatment-resistant depression with comorbid mental illness	Co-formulation may reduce pill burden but requires compatible drug dose, stability, release kinetics, and safety profiles	Initially use coordinated administration of separate products Pursue fixed-dose co-depots only when dose ratios and clinical rationale are stable	Co-encapsulated microspheres, ISFDs, and hybrid depots face the greatest challenge as separate LAIs are more controllable
Emerging materials	Candidates that cannot meet loading, stability, release, or tolerability targets using established platforms	Novel materials may improve release control or loading but introduce uncertainty regarding long-term safety, scalability, and regulatory acceptance	Demonstrate reproducible manufacture, long-term safety, predictable in vivo release, and clear advantage over established depot systems	Hydrogels, nanocomposites, and responsive systems may improve delivery but have limited clinical and regulatory precedent

## Data Availability

No new data were created or analyzed in this study. Data sharing is not applicable to this article.

## Abbreviations

The following abbreviations are used in this manuscript:

5-HT	5-Hydroxy Tryptamine or Serotonin
AI	Artificial intelligence
BBB	Blood–brain barrier
CNS	Central nervous system
ICHQ3C(R9)	International Council for Harmonisation guideline Q3C(R9): Impurities—Guideline for Residual Solvents
IM	Intramuscular
ISFDs	In situ-forming depots
IVIVC	In vitro–in vivo correlation
LAI	Long-acting injectable
MAOIs	Monoamine oxidase inhibitors
MDD	Major depressive disorder
ML	Machine learning
NE	Norepinephrine
NIR	Near-infrared
NLCs	Nanostructured lipid carriers
PBPK	Physiologically based pharmacokinetic
PCL	Polycaprolactone
PDE	Permitted daily exposure
PEG	Polyethylene glycol
PLA	Polylactic acid
PLGA	Poly(lactic-co-glycolic acid)
SC	Subcutaneous
SLNs	Solid lipid nanoparticles
SNRIs	Selective serotonin–norepinephrine reuptake inhibitors
SSRIs	Selective serotonin reuptake inhibitors
STARD	Sequenced Treatment Alternatives to Relieve Depression
TCAs	Tricyclic antidepressants
WHO	World Health Organization
